# MEF2C shapes the microtranscriptome during differentiation of skeletal muscles

**DOI:** 10.1038/s41598-021-82706-2

**Published:** 2021-02-10

**Authors:** Agnieszka Piasecka, Michał Sekrecki, Michał Wojciech Szcześniak, Krzysztof Sobczak

**Affiliations:** 1 Department of Gene Expression, Institute of Molecular Biology and Biotechnology, Faculty of Biology, Adam Mickiewicz University, Uniwersytetu Poznanskiego 6, 61-614 Poznań, Poland; 2 Institute of Human Biology and Evolution, Faculty of Biology, Adam Mickiewicz University, Uniwersytetu Poznanskiego 6, 61-614 Poznań, Poland

**Keywords:** Cell biology, Developmental biology, Molecular biology

## Abstract

Myocyte enhancer factor 2C (MEF2C) is a transcription factor that regulates heart and skeletal muscle differentiation and growth. Several protein-encoding genes were identified as targets of this factor; however, little is known about its contribution to the microtranscriptome composition and dynamics in myogenic programs. In this report, we aimed to address this question. Deep sequencing of small RNAs of human muscle cells revealed a set of microRNAs (miRNAs), including several muscle-specific miRNAs, that are sensitive to MEF2C depletion. As expected, in cells with knockdown of MEF2C, we found mostly downregulated miRNAs; nevertheless, as much as one-third of altered miRNAs were upregulated. The majority of these changes are driven by transcription efficiency. Moreover, we found that MEF2C affects nontemplated 3′-end nucleotide addition of miRNAs, mainly oligouridylation. The rate of these modifications is associated with the level of TUT4 which mediates RNA 3′-uridylation. Finally, we found that a quarter of miRNAs which significantly changed upon differentiation of human skeletal myoblasts is inversely altered in MEF2C deficient cells. We concluded that MEF2C is an essential factor regulating both the quantity and quality of the microtranscriptome, leaving an imprint on the stability and perhaps specificity of many miRNAs during the differentiation of muscle cells.

## Introduction

Myocyte enhancer factor 2 (MEF2) is a family of transcription factors important for the regulation of gene expression in many tissues, including the heart and skeletal muscles. These transcription factors play important roles in development, differentiation, regeneration and adaptation to a wide array of physiological and pathological signals. MEF2 proteins were identified as essential regulators of skeletal muscle-specific transcription and as partners for myogenic differentiation 1 (MYOD1) and other myogenic factors^1–3^.

In humans, there are four MEF2 paralogs (MEF2A, B, C and D). They share an N-terminal MADS domain and MEF-domain, which are responsible for protein dimerization and sequence-specific DNA binding^4,5^. MADS and MEF2 interact with a number of cofactors. In the C-terminal regions of MEF2 proteins, there are transcription activation domains, which are important targets for phosphorylation and other posttranslational modifications that regulate MEF2 function^6^. The C-terminal regions of MEF2 paralogs are highly divergent and highly variable, which is partially caused by alternative splicing^7–9^. The *MEF2C* gene consists of 13 exons, among which three are alternatively spliced, yielding many splicing isoforms^10^. In contrast to other paralogs, *MEF2C* is expressed in a tissue-specific manner and is restricted to the spleen, brain, and skeletal and cardiac muscles^11,12^. Expression analysis in adult mouse tissues revealed that the major *Mef2c* isoforms are differentially expressed in brain and skeletal muscles^8,9,13^. In summary, the foregoing analysis showed that due to the expression of distinct MEF2 paralogs and their splicing isoforms in different tissues and developmental stages, alteration in the expression of target genes can be observed.

An inherent transcription myogenic activity of MEF2 proteins can be modulated trough interaction with a diverse array of cofactors^1,14^,depending on the formed complexes, MEF2 proteins can function as either an activator or a repressor of gene transcription via chromatin remodeling^9^. MEF2s control the balance between chromatin acetylation and deacetylation and thereby regulate the relative accessibility of promoters and enhancers for the core transcriptional machinery^15–17^. Although many reports demonstrate the involvement of MEF2s in the regulation of protein-coding gene expression, there are only a few papers showing a regulatory role of these proteins in miRNA biosynthesis. Many miRNAs that are expressed in skeletal muscles remain under regulation of the myogenic program,on the other hand, these miRNAs themselves target a wide range of muscle-specific genes to coordinately control myogenesis. This group includes four miRNAs: miR-1, miR-133a, miR-133b and miR-206, known as myomiRs, organized in a genome as three bicistronic clusters. MiR-1 and miR-133a are expressed in both cardiac and skeletal muscles, while miR-206 and miR-133b are skeletal muscle-specific. A few years ago, it was reported that MEF2s directly regulate the expression of myomiRs through binding to a muscle-specific enhancer located within an intron separating the miR-1–2 and miR-133a-1 coding regions. A similar muscle-specific intragenic enhancer that controls transcription of the miR-1–1/-133a-2 *locus* was identified^18^. However, it was not determined which of the MEF2 paralogs is a major activator of miR-1 and miR-133a in vivo. More recently, it was demonstrated that in cardiac cells, the level of ten pri-miRNAs is MEF2C-sensitive^19^, but the effect of MEF2C on mature miRNAs was not studied. Because of differences in the expression pattern of MEF2 paralogs, MEF2C splicing isoforms and their interacting cofactors in heart and skeletal muscles, the combined effect of MEF2C on pri-miRNAs may differ significantly in these tissues.

The aim of our study was to explain the contribution of MEF2C activity to miRNA regulation during skeletal muscle differentiation. We performed deep sequencing of a small RNA pool in MEF2C-depleted myocytes to reveal quantitative and qualitative changes in microtranscriptome. We proposed pathways in which MEF2C may impact miRNA metabolism. Knock-down of MEF2C affected the expression of dozens of miRNAs, among which, two third were downregulated. We examined the mechanism underlying MEF2C-dependent regulation of miRNAs and we concluded that most of observed changes are transcription-dependent. Furthermore, in muscle cells with depletion of MEF2C we revealed the impairment of miRNA 3′-uridylation, what is associated with accumulation of short miRNA fragments perhaps as a consequence of their defective removal and as a result of repression of TUT4, a major terminal uridylyltransferase. Finally, we found that as many as a quarter of miRNAs which expression is significantly changed upon differentiation of muscle cells is MEF2C-sensitive. Our results demonstrated a significant role of MEF2C during the differentiation of skeletal muscles in sculpting the microtranscriptome in both transcriptional and posttranscriptional miRNA biogenesis pathways.

## Results and discussion

### Depletion of MEF2C disrupts expression of several miRNAs in differentiated muscle cells

**Figure 1:**
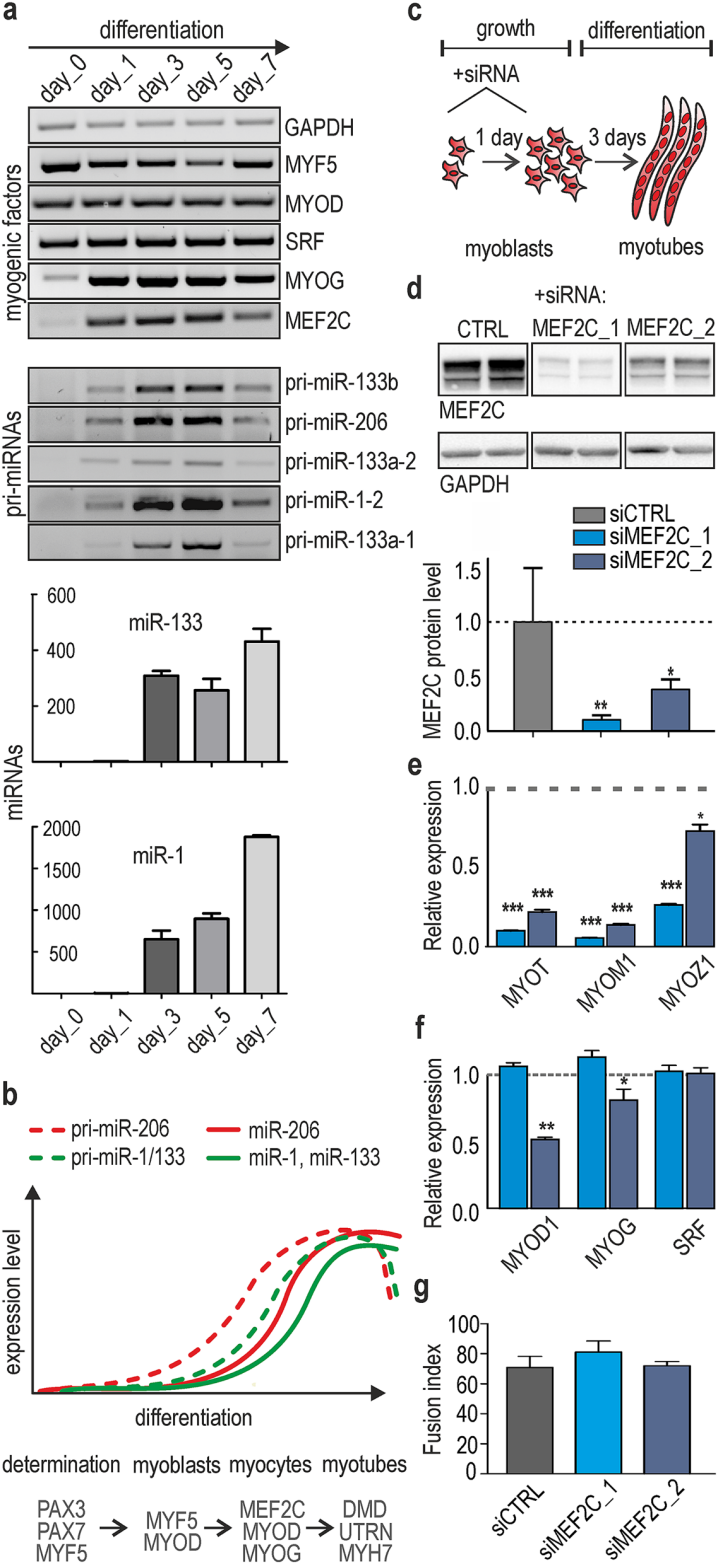
The expression of muscle-specific miRNAs is correlated with MEF2C and MYOG level during skeletal muscle differentiation. (**a**) RT-PCR-based quantification of the level of miRNA, pri-miRNAs and mRNA of myogenic factors performed for primary human myoblasts in growth (day 0) or differentiation conditions (days 1, 3, 5 and 7). Image was created by using GeneTools 4.3.9.0 and CorelDRAW 2017. Fully uncut gel images are shown in Supplementary information, uncut images section; level of miR-1 and miR-133 was analyzed using TaqMan real-time PCR normalized to U6RNA. The three human primary myoblasts were tested. (**b**) Graph showing the pattern of temporal expression changes in selected pri-miRNA and their mature forms. Specific markers of different stages of muscle cells differentiation are shown above the graph. (**c**) Experimental timeline for the HSkM cell cultures. Proliferating HSkM cells were transfected with siCTRL, siMEF2C_1 or siMEF2C_2 (day 0) and then transferred to differentiation medium. The next day, the cells were transfected once again and cultured in differentiation medium for the next 3 days. (**d**) The steady state level of MEF2C protein (normalized to GAPDH) was strongly reduced during the course of the experiment. siMEF2C_1 induced more efficient reduction of MEF2C. The experiment was performed in HSkM cells in triplicates. Signals were quantified with GeneTools from Syngene (https://www.syngene.com/software/genetools-automatic-image-analysis/)**.** Fully uncut gel images are shown in Supplementary information, uncut images section. (**e**) Three MEF2C-dependent target genes encoding muscle-specific proteins subsequently showed reduced expression on mRNA level. (**f**) The main markers of myogenesis (MYOD, MYOG and SRF) were not down-regulated in differentiated HSkM cells treated with siMEF2C_1; moderate decrease of MYOD and MYOG was observed in siMEF2C_2 KD experiments. The results in (**e**) and (**f**) are average results of RT-qPCR analyses performed for three independent samples from siMEF2C-treated (blue bars) and siCTRL-treated cells (gray bars) normalized to GAPDH. Standard deviation (SD) and statistical significance based on an unpaired Student’s t-test are shown (* *P* < 0.05, **P < 0.01, ***P < 0.0001). (**g**) Fusion index of HSkM was not changed in differentiated myoblasts treated with siMEF2C_1 or siMEF2C_2. The fusion index was calculated as the number of nuclei within myotubes expressed as a percentage of the total number of nuclei in the image frame. Calculations were performed for three independent samples from siMEF2C_1 or siMEF2C_2-treated (blue bars) and siCTRL-treated cells (gray bar).

Myoblast differentiation is a multistep process that is governed by a complex network of myogenic factors, mainly myogenic differentiation 1 (MYOD1), myogenic factor 5 (MYF5), myogenin (MYOG), serum response factor (SRF), MEF2s and a number of myomiRs. To determine which myogenic factors exhibit similar expression patterns as myomiRs, we analyzed their levels during the differentiation of several cultures of primary human myoblasts. In all tested primary myoblasts cultures, we observed a strong positive correlation between the expression of muscle-specific miRNAs and the steady state level of MEF2C as well as MYOG, which was previously confirmed to be an important myomiR regulator^20,21^ Fig. 1a,b. More importantly, MEF2C showed the most abrupt and steep increase of expression level at the same window time as myomiRs (Fig. S1a). The expression of other myogenic factors (MYOD, SRF, MEF2A and MEF2D) was already observed at earlier stages of differentiation. We decided to track microtranscriptome changes related to MEF2C and asked whether it is an important driver of miRNA biogenesis in muscle cells.

To answer this intriguing question, we used differentiating human skeletal myoblasts (HSkM cells) with or without knock-down of *MEF2C*. Since differentiated cells are hard to transfect, control siRNA (siCTRL) and two different siRNAs against MEF2C (siMEF2C) were delivered twice into proliferating myoblasts, and then the cells were allowed to differentiate for the next 3 days Fig. 1c. This protocol allowed us to efficiently knock-down *MEF2C* expression (MEF2C KD). We monitored an efficiency of knock-down in the timeframe. MEF2C KD was very effective in days 3 and 4 of differentiation, thereon MEF2C level started to restore (Fig. S1b). Therefore, we decided to analyze the effects of MEF2C KD in day four, when high percentage of cells is fused and form myofibers. We used two different siRNAs against MEF2C: (i) previously published siRNA targeting a single sequence (^22^ Supplementary Table S1), further referred to as siMEF2C_1, and (ii) SMARTpool siRNA which combines four MEF2C-specific siRNAs, further referred to as siMEF2C_2. siMEF2C_1 was more effective and knock-down *MEF2C* to ~ 10% at the mRNA and to 15% at protein level, whereas siMEF2C_2 knocked-down *MEF2C* to ~ 30% at the mRNA and at 35% of protein level Fig. 1d. To determine if MEF2C KD was sufficient to cause downstream effects, we also checked the level of three known MEF2C target genes encoding myozenin, myomesin and myotilin. We observed an expected reduction in their steady state levels for both siRNAs Fig. 1e. We also monitored the dynamics of cell differentiation by microscopy and noticed no disruption of myotube formation in MEF2C-depleted cells. Furthermore, in siMEF2C_1 treated cells there was no significant decline in mRNA level of other important myogenic factors, e.g., SRF, MYOD1, MYOG , MEF2A or MEF2D Fig. 1f and Fig. S1c), and we did not observe significant decrease of MYOG protein level (Fig. S1d). Additionally, the fusion index of HSkM myotubes in the presence of siCTRL or siMEF2C_1 or siMEF2C_2 was calculated and we did not observe significant differences in the percent of nuclei within myotubes Fig. 1g. We speculated that the amount of remaining MEF2C protein in siRNA treated cells may be sufficient to orchestrate a phenotypic differentiation program. Upon treatment with siMEF2C_2 a moderate reduction of *MYOD1* and *MYOG* expression level was noticed. Therefore, in further studies data obtained after siMEF2C_1 treatment were considered as more precise and clear-cut (not burdened with the consequences of *MYOD1* and *MYOG* expression changes).

**Figure 2:**
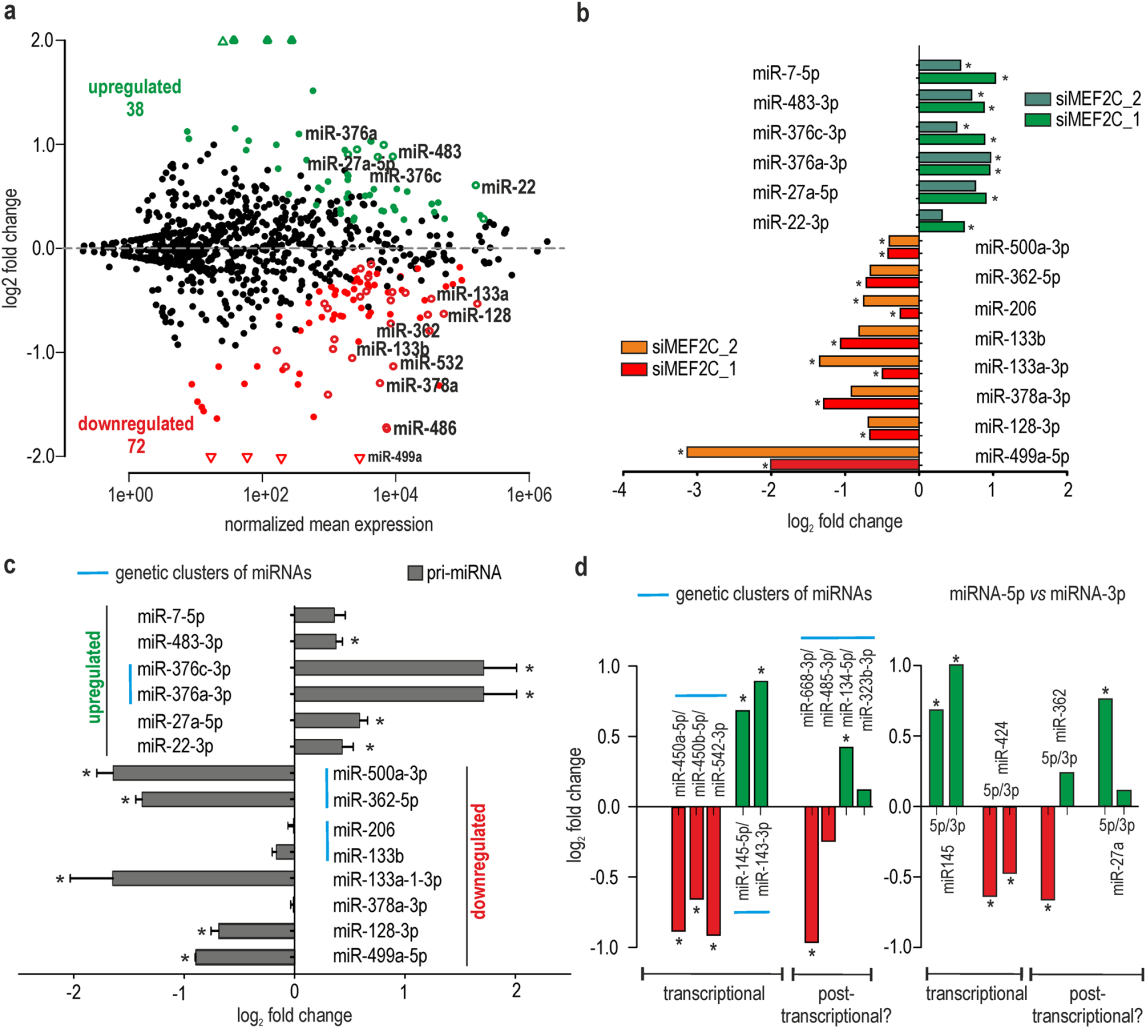
Changes in the expression profile of miRNAs in human skeletal myocytes with MEF2C depletion. (**a**) MA-plot showing the prediction of differentially expressed miRNAs between cells treated with control and MEF2C-specific siRNAs. The red solid dots represent the statistically significant results for miRNAs downregulated in cells treated with siMEF2C_1; the green solid dots represent significantly upregulated miRNAs by siMEF2C_1. Empty dots represent miRNAs deregulated by both siMEF2Cs (_1 and_2). The experiment was performed in HSkM cells in triplicates. (**b**) Validation of the results from (**a**) in HSkM with MEF2C KD. The expression of 14 mature miRNAs was quantified using polyA-RT-qPCR analysis. The expression level of miRNAs were normalized to the level of the small nuclear U6RNA. Green/dark green and red/orange bars represent miRNAs predicted to be upregulated or downregulated according to RNA-seq after treatment with siMEF2C_1 or siMEF2C_2, respectively. The results are average values from three independent experiments + / − SD (* *P* < 0.05). (**c**) Relative expression changes of pri-miRNAs quantified by RT-qPCR assays divided into two groups (miRNAs predicted to be upregulated or downregulated according to RNA-seq results); all data for pri-miRNA were averages from three independent experiments + / − SD normalized to mRNA of GAPDH (* *P* < 0.05). MiRNAs from the same genetic cluster are marked with a blue line. (**d**) Expression changes of miRNAs coming from the same pre-miRNA hairpin or from the same genetic cluster (marked with blue lines) based on RNA-seq data. MiRNA candidates which could be regulated transcriptionally or posttranscriptionally are indicated.

In the next step, we sequenced a pool of small RNA fractions (small RNA-seq) isolated from MEF2C KD myoblasts using the NovaSeq 6000 system (Illumina, USA). Plain miRNA counts were identified from the small RNA-seq input samples and mapped against all known human miRNA precursor sequences deposited in the miRBase database. It is well proven that many miRNA genes give rise to a population of heterogeneous miRNA products that have variable sequence lengths at both ends^23–25^. Thus, a mature miRNA sequence was redefined to not be restricted to one canonical, miRBase-annotated sequence. In the modified analysis, we allowed for up to 2 nt longer, templated, and 2 nt shorter isoforms (± 2 nt). The average number of sequencing reads for control cells was 45,181,500, the average number of sequencing reads for siMEF2C_1 and siMEF2C_2 treated cells was 42,675,000 and 39,035,000 respectively. Over 550 mature miRNAs were identified in all tested samples (a cutoff of a baseMean > 10,the mean of normalized counts of all samples, normalizing for a sequencing depth greater than 10), among which as many as 120 were predicted to be differentially expressed (*P*_adj_ < 0.05; 24 differentially expressed if *P*_adj_ < 0.01) between control and siMEF2C_1 KD cells (Supplementary information 1). In siMEF2C_2 KD cells, probably due to the less efficient silencing of MEF2C, changes were not that deep; the number of differentially expressed miRNA, at the same boundary conditions, reached 54 (Supplementary information 2). 70% of differentially expressed miRNA in siMEF2C_2 KD cells were also significantly changed upon siMEF2C_1 knock-down. In both sequencing experiments, two third of deregulated miRNAs were predicted to be downregulated (82 out of 129 and 45 out of 65, respectively). However, it is worth noticing that a significant fraction of miRNAs had a considerably elevated expression Fig. 2a.

To validate sequencing data, we chose 14 miRNAs which were significantly deregulated in both data sets and we performed poly(A) tailing-based RT-qPCR, which allowed the quantification of nearly all sequence variants for each miRNA. Briefly, the total RNA was polyadenylated with *E. coli* poly(A) polymerase and converted into cDNA in a reverse transcription reaction primed by an oligo-dT anchor adaptor. The level of miRNAs was then analyzed by real-time PCR using a miRNA-specific forward primer and a universal adaptor-specific primer^26^. All tested miRNAs were positively validated in at least one of cellular models, either siMEF2C_1 or siMEF2C_2 treated myocytes and 70% of miRNAs (10 out of 14) were validated in both of them Fig. 2b. We noted higher validation of RNA-seq results in case of siMEF2C_1 KD and we presume that this effect was partially a result of more efficient silencing of *MEF2C*.

To determine whether identified changes in miRNA levels are driven by transcriptional or posttranscriptional mechanisms, we measured the level of corresponding primary miRNAs (pri-miRNA). The expression level of the majority of mature miRNAs (10 out of 14) positively correlated with the level of pri-miRNA forms Fig. 2c. Upon MEF2C decline a transcriptional repression of many miRNA genes, including myomiRs from the miR-1–2/133a-1 genetic cluster, but not the miR-206/133b and miR-1–1/133a-2 clusters was observed Fig. 2c and Fig. S2a). We also identified a few miRNAs which were transcriptionally activated in MEF2C deficient myoblasts. However, we cannot rule out that upon MEF2C deficiency, the expression of some of these miRNAs increased as a competition effect between different MEF2 paralogs for which target miRNA genes can overlap, as it is observed for target protein-encoding genes^27^. Among MEF2C-sensitive miRNAs we distinguished a group of miRNAs maturated from the same genetic clusters, what suggests their common expression regulation. Beside above mentioned one myomiR cluster, this group also includes miR-145/143 and miR-450a/450b/542 clusters Fig. 2d. To probe whether MEF2C could directly regulate part of deregulated miRNAs, we searched 1 kb genomic regions spanning each of the top 16 differentially expressed miRNAs in MEF2C deficient myocytes for MEF2 binding sites using *Pscan* programme^28^ (Table S7). In all of them we found putative MEF2 binding sites. The results suggest that the listed miRNAs might be directly regulated by MEF2C by binding to their promoters. Any of available algorithms take into account all aspects of MEF2 binding mechanism, which go beyond recognizing the nucleotide identity of each individual position within the binding site^29^ .Nevertheless, the predicted binding sites are scored higher than validated for miR-133a-2 gene. Based on obtained results it can be assumed that silencing of *MEF2C* affects transcription of numerous miRNA genes. However, we cannot distinguish whether it is direct or indirect effect or it depends on feedback mechanisms, secondary targets or related myogenic proteins influenced by MEF2C ablation.

For some miRNAs (4 out of 14), the RT-qPCR results for pri-miRNAs and miRNAs did not correlate well Fig. 2c and Fig. S2a). This could be caused by either the occurrence of several different genomic *loci* for the same miRNA or posttranscriptional RNA processing, such as modification of pre-miRNA or mature miRNA sequences, affecting efficiency of miRNA biogenesis or turnover. The evidence of posttranscriptional regulation could involve a divergent level of miRNAs coming either from the same pre-miRNA hairpin, e.g., miR-362-5p/3p, miR-181a-5p3p and miR-675-5p/3p or from the same genetic cluster, e.g., miR-668/485/134/323b, miR-487b/381 and miR-25/93 clusters Fig. 2d and Fig. S2b,c).

### MEF2C tunes up oligouridylation of many miRNAs

**Figure 3:**
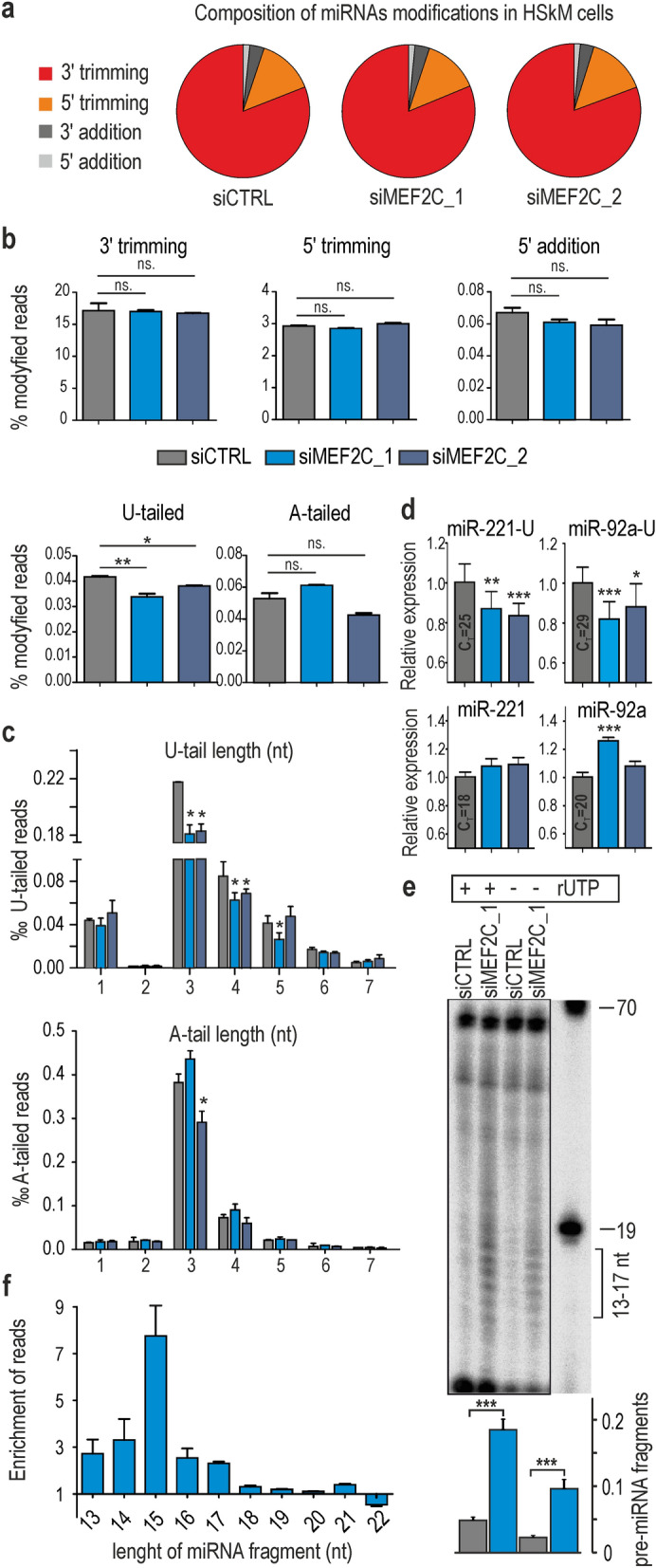
MEF2C regulates 3′-uridylation of miRNAs in human muscle cells. (**a**) The pie charts show the frequency of four types of modifications of the miRNA sequence in differentiated HSkM cells. The experiment was performed in HSkM cells in triplicates. (**b**) Comparison of the frequency of 5′- and 3′-modifications of miRNAs in control and MEF2C KD cells based on the results of small RNA-seq experiments ( *P < 0.05; **P < 0.01). (**c**) Comparison of the number and length of 3′-nontemplated U- and A-tailed reads of miRNAs in control and siMEF2C_1 or siMEF2C_2 treated myocytes. The X-axis represents the length of U- or A-tails, and the Y-axis represents the percentile of modified reads in small RNA-seq ( *P < 0.05). (**d**) Modified polyA-RT-qPCR assays were used to quantify canonical forms of miR-221 and miR-92a (lower panels) and uridylated isoforms of miR-221-U and miR-92a-U (upper panels), in muscle cells with MEF2C deficiency (*P < 0.05, **P < 0.01, ***P < 0.0001). The experiment was performed in triplicates. (**e**) In vitro uridylation assay of internally radiolabeled pre-miR-27b incubated with extracts from siCTRL- or siMEF2C_1-treated HSkM cells. The mixtures were either supplemented or not supplemented with 0.25 mM UTP (+ or − rUTP). Reaction products were separated by denaturing PAGE, analyzed by autoradiography, and the amount of pre-miRNA short fragments (13–17 nt) was calculated and is shown on a graph (below) as a fraction of unprocessed pre-miRNA (***P < 0.0001). Fully uncut gel images are shown in Supplementary information, uncut images section. (**f**) Accumulation of short fragments of miRNAs in differentiated HSkM cells upon siMEF2C_1 KD calculated based on RNA-seq data (n = 3 for siCTRL and n = 3 for siMEF2C_1). Alterations in the level of miRNA fragments are shown as fold changes; fold change is defined as the ratio between the counts of short fragments of miRNA in siMEF2C_1 KD and the counts of short fragments of miRNA in siCTRL cells divided by the counts of short fragments of miRNA in siCTRL cells (no change is equal to 1).

In view of the foregoing results, it seems that MEF2C does not only impact miRNA expression level via transcription regulation. Hence, we decided to examine another layer of regulation which is miRNA’s modifications including most common non-templated additions and trimmings at miRNA’s ends. To describe the global range of changes in miRNA isoform distribution, we used miRMode, a program that identifies 5′- and 3′-modifications of analyzed sequences in small RNA-seq data. This analysis revealed trimming of miRNA 3′-ends as the most frequent modification in differentiated HSkM cells, followed by 3′-addition, 5′-trimming and 5′-addition Fig. 3a. Observed distribution of miRNA modifications resembled the one noted in other types of mammalian cells^30^. Although we did not observe any significant differences in 3′- or 5′-trimming or 5′-addition between control and MEF2C KD cells, we did notice a modest but significant reduction in 3′-nontemplated nucleotide additions (3′-NTAs). Having examined two most common types of 3′-NTAs, namely adenylation and uridylation, we observed two regularities: first, upon treatment of both siMEF2C_1 and siMEF2C_2, the expression level of U-tailed, but not A-tailed miRNAs was considerably decreased Fig. 3b. Second, the length distribution of additional short uridine tracks, with a predominance of 3 nt-long U-tails, was not disturbed in MEF2C KD. It seems that MEF2C affects oligouridylation rather than monouridylation Fig. 3c. Since, the observed decline in uridylation efficiency was modest, we decided to repeat the experiment with another protocol of small RNA libraries preparation. We used TruSeq Small RNA kit (Illumina) for generation siCTRL and siMEF2C_1 libraries. This approach revealed much deeper alternations in oligouridylation upon MEF2C depletion (Fig.S5a). To confirm a decrease of oligouridylation upon MEF2C KD we used a modified protocol of the poly(A) tailing-based RT-qPCR method so that a forward primer preferentially bound to uridylated miRNA isoforms. We were able to increase selectivity of the primers by the addition of three T-residues at the 3′-end (Fig. S3). Attained results confirmed a decline of miRNA oligouridylation,we noticed significant decrease of miR-92a and miR-221 uridylated isoforms in relation to canonical miRNAs Fig. 3d.

To determine whether MEF2C-dependent miRNA 3′-additions occur initially on precursor forms of miRNAs, we analyzed mature miRNA sequences derived from the 5p- and 3p-arms of pre-miRNA hairpins (miRNA-5p and miRNA-3p, respectively) (Fig. S4 and S5b). We assumed that all modifications that occur on the 3′-end of pre-miRNA can be observed at the 3′-end of mature miRNA-3p but not miRNA-5p. Obtained results revealed that in general in muscle cells the uridylation was approximately ten times more frequent in miRNAs derived from the 3′-arm of pre-miRNA (Fig. S4, left panel). In contrast, adenylation, the most frequent tailing event, was similar between miRNA-5p and miRNA-3p and thus most likely occurred on mature miRNAs. In MEF2C KD cells the percentage of oligouridylated miRNAs was significantly lower only for miRNA-3p. Therefore, we concluded that MEF2C-dependent U-tailing occurs mostly on pre-miRNAs.

To gain further insight into the MEF2C-dependent uridylation of pre-miRNA, we performed an in vitro uridylation assay in total extracts from normal and MEF2C KD cells Fig. 3e. The most striking observation was the accumulation of short RNA fragments (13–17 nt) of two tested pre-miRNAs Fig. 3e. To determine whether the accumulation of short miRNA fragments occurs also in vivo*,* we analyzed modifications of truncated miRNA sequences in small RNA-seq data using the miRMode program. Short miRNA fragments (13–17 nt) were found to be up to 6-times enriched in siMEF2C_1 KD cells and up to 2-times enriched in siMEF2C_2 KD cells Fig. 3f and Fig. S5c).

All these results suggest that the MEF2C depletion leads to decreased steady state level of oligouridylated miRNAs, what could be a consequence of either lower RNA uridylation or higher degradation rate of uridylated miRNAs. Moreover, insufficiency of MEF2C increases the level of short pre-miRNA fragments. This effect can be a consequence of a higher degradation pace of miRNAs or their precursors or stabilization of intermediates of pre-miRNA turnover.

### MEF2C contributes to the miRNA surveillance pathway by affecting TUT4 activity

Addition of nontemplated nucleotides is driven by terminal nucleotidyl transferases (TNTs), which typically add adenosine or uridine residues to 3′-ends. Recent studies have shown that these enzymes are involved in controlling every aspect of miRNA lifespan, including maturation, stability, silencing, sorting into exosomes and degradation. In humans, there are at least three enzymes that have the potential to add non‐templated U-residues to the 3′-end of pre-miRNAs or mature miRNAs. These enzymes are known as terminal uridyl transferases (TUTases; TUT2, 4 and 7). It was further reported that the nucleotide addition by these enzymes is miRNA-specific.

**Figure 4:**
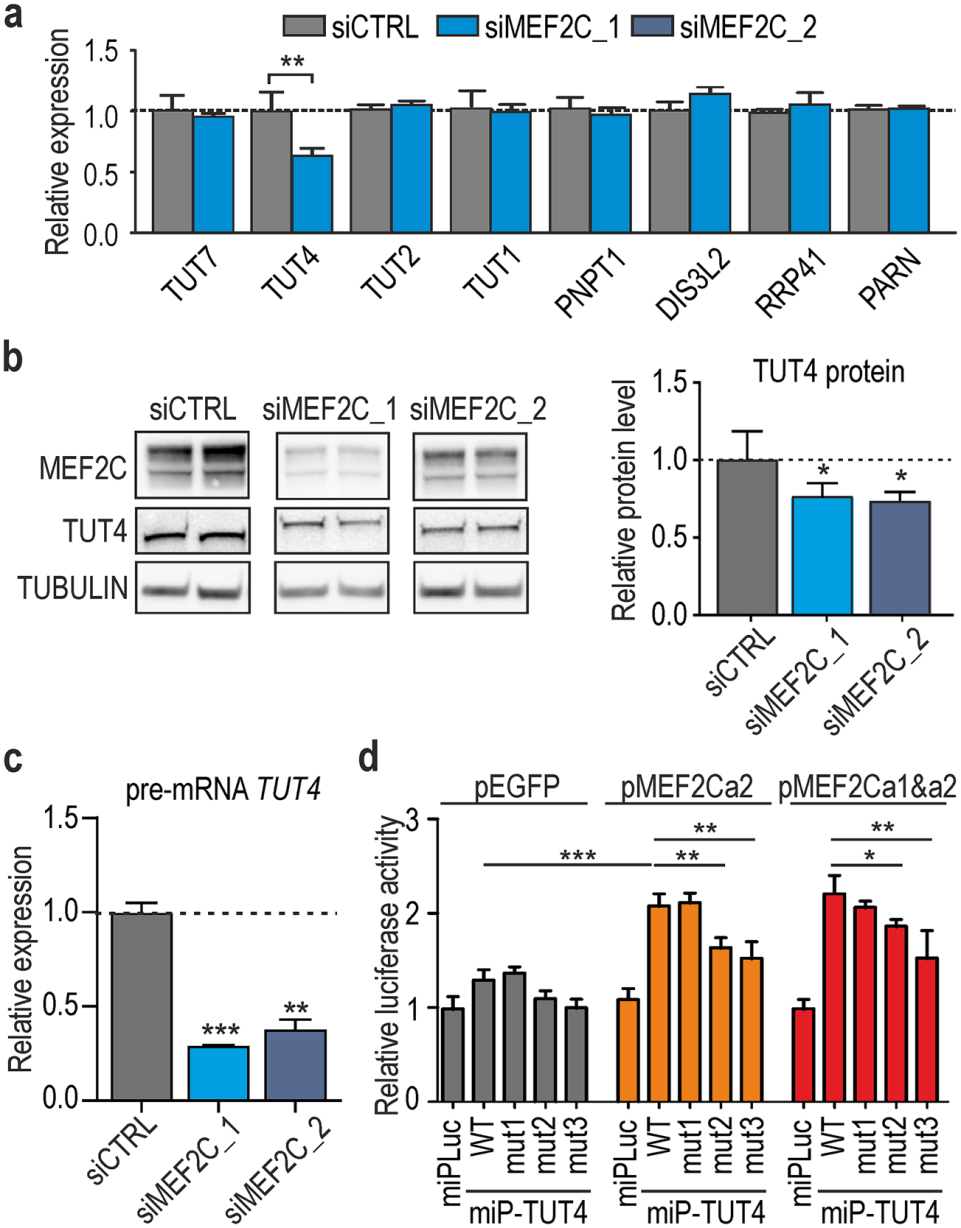
MEF2C contributes to the 3′-uridylation of miRNAs via TUT4 activity. (**a**) Results of RT-qPCR for mRNAs encoding for TUTs and other proteins related to the turnover of miRNA (PNPT1, polyribonucleotide nucleotidyltransferase 1; RRP41, ribosomal RNA-processing protein 41; PARN poly(A)-specific ribonuclease). Each bar represents average from three independent biological replicats + / − SD normalized to GAPDH (**P < 0.01). (**b**) Reduction in the level of TUT4 protein in MEF2C KD conditions confirmed by western blotting. Quantification of TUT4 is depicted in the graph below and based on 3 independent experiments normalized to GAPDH (*P < 0.05). Signals were quantified with GeneTools from Syngene (https://www.syngene.com/software/genetools-automatic-image-analysis/)**.** Fully uncut gel images are shown in Supplementary information, uncut images section. (**c**) RT-qPCR analysis of pre-mRNA of TUT4. The amplified region was located in intron 2 of *TUT4*. The results are averages from 3 independent experiments normalized to GAPDH mRNA (= / − SD). The *P* value was assessed by an unpaired *t*-test (**P < 0.01; ***P < 0.001). (**d**) Normalized luciferase activity calculated for HeLa cells cotransfected with genetic constructs expressing two isoforms of MEF2C (MEF2Ca1 and MEF2Ca2) or GFP and the *LUC* gene with either a control minimal promotor (miPLuc) or minimal promotor of *LUC* with upstream fragment of *TUT4* gene (miPfTUT4) or minimal promotor of *LUC* with upstream fragment of TUT4 gene with mutations of putative MEF2C binding sites (miPmut1TUT4 or miPmut2TUT4 or miPmut1TUT4; see Table S8 for more details). Sequence of minimal promotor is depicted in Supplementary Table S2. The experiment was performed in 5 replicates, and the *P* value was assessed by an unpaired Student’s t-test (*P < 0.05; **P < 0.01).

Described above results of our study suggest that MEF2C increases the level of 3′-uridylated miRNAs what could be a result of either lower activity of enzymes responsible for degradation of uridylated miRNAs or higher activity of TUTases, which mark miRNA and their degradation intermediates. Therefore, we examined the expression levels of several TUTases and other proteins responsible for miRNA turnover^31,32^. Among the eight tested enzymes, only one, TUT4, which is involved in miRNA uridylation, was decreased by approximately 30–40% at mRNA and protein level upon treatment of cells with siMEF2C_2 or siMEF2C_1 Fig. 4a,b and Fig. S6a,b). The obtained results suggest that MEF2C could be responsible for the regulation of TUT4 expression and consequently affect the miRNA surveillance pathway. To test how reproducible the effects are in other cell line, we investigated changes in the expression of TUT4 and uridylation of four miRNAs in differentiated C_2_C_12_ mouse myoblasts under depletion of Mef2c. We knock-downed *Mef2c* using siMEF2C_2 and used the same protocol as for HSkM cells . We observed TUT4 decrease by approximately 40% at mRNA and 30% at protein level (Fig. S7a and S7b). The decrease of U-tailed isoforms of miR-92a, miR-148b and miR-221 in C_2_C_12_ cells was similar as in HSkM cells (Fig. S7c).

Since MEF2C is involved in transcriptional regulation of numerous genes, we considered a direct control of *TUT4* gene by MEF2C. To investigate this possibility, we first compared the temporal expression of *TUT4* and *MEF2C* at the mRNA level. The expression of both genes was positively correlated during muscle cell differentiation; however, the increase of TUT4 level upon differentiation was significantly lower than that of MEF2C (Fig. S6c and Fig. S8b). To distinguish between transcriptional and posttranscriptional regulation of TUT4, we examined whether its decrease is observed already at pre-mRNA level. The results of RT-qPCR analysis indicated that there is significant decrease of TUT4 pre-mRNA level Fig. 4c and Fig. S6d). Thus, the TUT4 decline observed upon MEF2C KD was at least partially a result of a transcriptional mechanism. Then, we searched for MEF2C binding sites within a 1 kb genomic region upstream to the *TUT4* gene using Pscan program^28^. We identified three putative MEF2C-binding sites within the ~ 1 kb of potential promoter sequence and tested activity of this region in a luciferase assay. We noticed that the studied *TUT4* promoter region was sensitive to MEF2C in two tested cell lines (the number of replicates, n = 5, Fig. 4d HeLa and Fig. S6e, COS7). Deletion of putative MEF2C binding sites resulted in diminished luciferase activity for two sites Fig. 4d. This finding implies that an increase of TUT4 during muscle differentiation is most likely a direct effect of higher MEF2C transcriptional activity.

Oligouridylation is the important modification of 3′-ends of miRNA which affects its activity. It is known that some miRNAs with U-tails are less stable compared to canonical miRNAs and miRNAs containing other modifications^33,34^. However, it should be kept in mind that different miRNAs differentially acquire terminal uridylation and then differentially respond to this modification. Foregoing research suggested substantial independence of U-tailing and degradation pathways with of mature, full-length miRNAs^30,33^. It was reported that deficiencies of TUT4 and TUT7 have only a minor effect on global miRNA expression levels and proposed that TUT4/7-mediated RNA 3′-uridylation functions rather in managing somatic cell stress responses or developmental processes than in RNA degradation in the steady state level^35^. It remains to be revealed whether muscle differentiation is one of these processes and can be partially regulated by MEF2C sensitive uridylation. Our data also indicate that MEF2C is able to reduce the number of shorter pre-miRNA fragments most likely by triggering their uridylation which marks them for removal Fig. 3e. These results are consistent with previous findings which show that in TUT7/4/2-depleted cells, the number of 3′-truncated fragments of pre-miRNA increases^36^.

Despite of miRNA activity, oligouridylation affects many other aspects of the miRNA lifespan including maturation, the efficiency of silencing and sorting into exosomes^36–39^. It was shown that miRNA uridylation can change the repertoire of mRNA targets. A target site that is normally nonfunctional because of the absence of a seed pairing match becomes functional when an upstream adenosine in the target site within mRNA base-pairs to the nontemplated U-tail of miRNA^40^. Therefore, miRNA specificity can also be regulated by MEF2C. Although mature miRNAs can serve as substrates for TUTases (44), our data suggest that MEF2C-sensitive oligouridylation occurs on pre-miRNA. Steady state level of miRNAs with nontemplated oligoU-tails is low, about 0.04% of all miRNA reads^30^,however, the conversion to U-tailed miRNA isoform may affect its stability. Otherwise, significance of this modification seems to be related not only to frequency of uridylation,uridylation was reported as crucial process in governing fate of selected miRNAs. The importance of uridylation for muscle differentiation is still unknown and remains to be revealed whether a decrease of U-tailing of miRNA and other RNA species affects muscle formation or function.

### MEF2C is an important player in miRNA dynamics during differentiation of muscle cells

**Figure 5:**
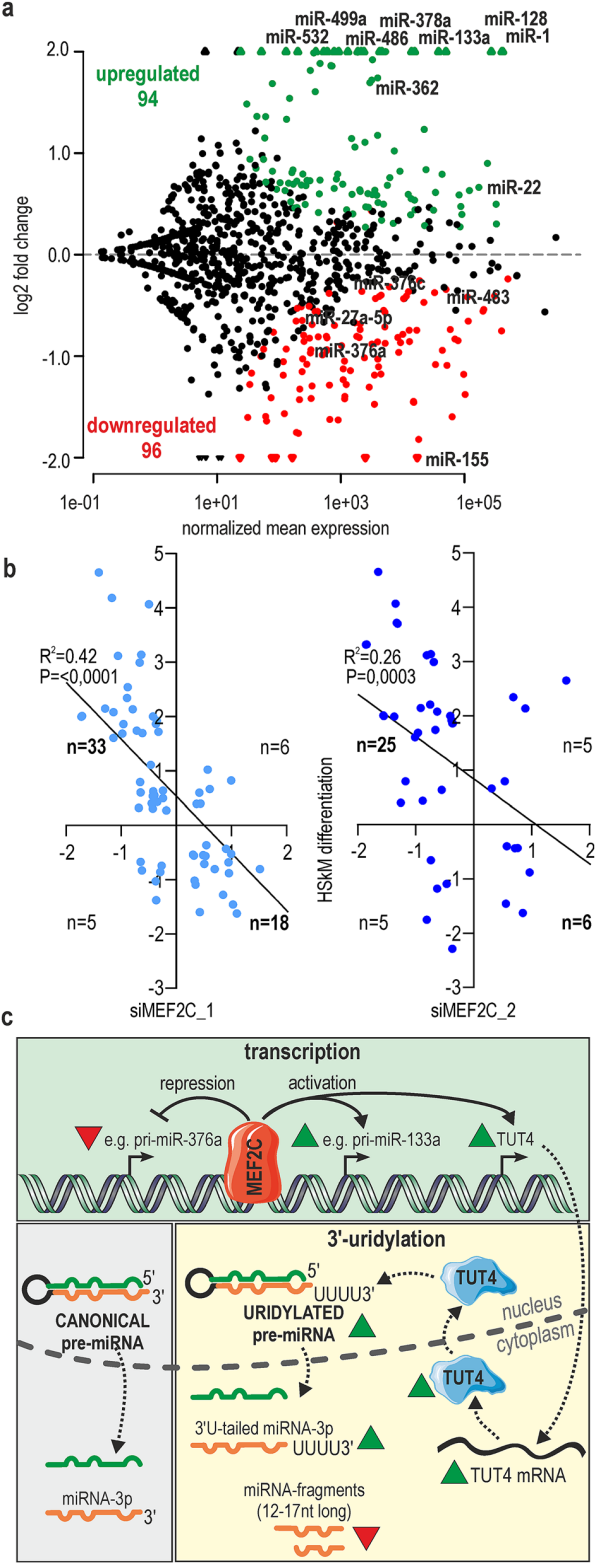
Contribution of MEF2C to miRNA regulation in differentiated muscle cells. (**a**) MA-plot showing the prediction of differentially expressed miRNAs between proliferating HSkM cells and myotubes after 4 days of differentiation (n = 3 HSkM_0h and n = 3 HSkM_4d). The red dots represent the statistically significant results for miRNAs downregulated; the green dots represent significantly upregulated miRNAs (*P*_*adj*_ < 0.05; a cutoff of a baseMean > 10). (**b**) Comparison of miRNA expression changes in two sets of experiments: MEF2C KD cells versus control treated cells (separately for siMEF2C_1 and siMEF2C_2) and 4 days differentiated muscle cells versus myoblasts (HSkM differentiation). Linear regression scatter plots of miRNAs log 2FC expression values were calculated by comparison of miRNAs significantly changed upon MEF2C KD (P_adj_ < 0.05) and miRNA significantly changed upon HSkMs differentiation (P_adj_ < 0.05). The statistical analysis for linear regression was done by using the GraphPad Prism 8.3.0 tool. (**c**) Schematic overview of MEF2C activity in muscle cells. MEF2C, as a transcription factor, can both activate (e.g., pri-miR-133a) and repress (e.g., pri-miR-376a) miRNA expression. Interaction of MEF2C within promoter of *TUT4* leads to increase of TUT4 level and normal 3′-uridylation of pre-miRNAs and miRNAs. Proper dynamics of uridylation guarantee the correct turnover of short pre-miRNA fragments. Red triangles represent downregulation, and green triangles upregulation.

Since the MEF2C is an important factor involved in myogenesis, we decided to determine its contribution to microtranscriptome changes during differentiation of HSkM. We sequenced small RNA fraction isolated from both proliferating HSkM cells cultured in growth medium (HSkM_0h; in triplicates) and HSkM cells after 4 days of differentiation (HSkM_4d; in triplicates), using the same protocol as in experiments with siMEF2C. Altogether, about 1,000 miRNAs were detected in these experiments, 612 of which were used for differential expression analyses (a cutoff of a baseMean > 10; the mean of normalized counts of all samples, normalizing for a sequencing depth greater than 10). 190 miRNAs were significantly changed between day 0 and day 4 of myoblast differentiation (*P*_adj_ < 0.05). 94 of them were upregulated and 96 downregulated Fig. 5a, Supplementary information 3). Taking into account highly expressed miRNAs (baseMean > 10,000), the highest upregulation was observed for miR-133a and miR-1 (myomiRs) and highest downregulation for miR-155-5p (engaged in muscle regeneration;^41^. We found that among 190 miRNAs significantly changed upon differentiation of HSkM cells as many as 63 and 41 miRNAs were significantly missregulated in cells treated with siMEF2C_1 and siMEF2C_2, respectively (Fig. S8a). Interestingly, direction of changes in MEF2C KDs (51 in siMEF2C_1 and 31 in siMEF2C_2) were inversely correlated with direction of miRNA changes upon HSkM differentiation, with a moderate coefficient of determination Fig. 5b. We assumed that a quarter of miRNA changes observed during myogenesis can be at least partially driven by MEF2C activity.

Our study showed the significance of MEF2C in the regulation of microtranscriptome dynamics in skeletal muscle differentiation Fig. 5c. Expectedly, we observed a positive correlation between expression of many miRNAs and the level of MEF2C. Nearly all tested miRNAs positively regulated by MEF2C have also been changed at pri-miRNA level implying that MEF2C regulates their transcription. On the other hand, about 20% of MEF2C-sensitive miRNAs could be regulated posttranscriptionally. MEF2C contributes to pre-miRNA 3′-oligouridylation through activation of *TUT4*. Finally we found that up to a quarter of miRNAs which expression changed during differentiation of myoblasts can be caused by increased activity of MEF2C. Summing up, a MEF2C governing role is important not only for the quantitative regulation of several miRNAs but also for the qualitative control of the microtranscriptome, leading to differences in their regulatory potential.

## Materials and methods

### Cell culture and transfection

Primary myoblasts (9905, 9936, 9206) were ordered from *Telethon Network of Genetic Biobanks* (TNGB) and they originated from skeletal muscle tissue biopsies. HSkM cells (Primary Normal Human Skeletal Myoblasts) were purchased from Gibco (Thermo Fisher).Myoblast cells were grown in HAM F-10 medium (Lonza) supplemented with 20% FBS (BioWest), 2 mM l-glutamine, 100 U/ml penicillin and 100 μg/ml streptomycin (Invitrogen) at 37 °C, 5% CO_2_. Upon shifting to a 2% horse serum-containing medium, HSkM myoblasts were induced to differentiate and fuse. The media were changed every day. To knock down MEF2C (MEF2C KD), the HSkM and C2C12 cells were transfected twice, in day 0 and day 1 of differentiation, using Lipofectamine 3000 (Invitrogen) according to the manufacturer's protocols; 50 nM siRNA was used. For the luciferase assays, HeLa or COS7 cells were transfected in 96-well plates at ∼70% confluence using Lipofectamine 3000 (Invitrogen) according to the manufacturer's protocols. For each transfection experiment, 100 ng of the appropriate reporter construct and 100 ng of the miPGLO-TUT4 vector or control vector were used. The cells were harvested 48 h after transfection and assayed for luciferase activity using a standard protocol (Dual-Glo Luciferase Assay System).

pEGFP-MEF2C were prepared in our laboratory. MEF2C cDNA was cloned into MCS of pEGFP-C1 plasmid. miPGLO plasmids were constructed by removing luciferase promoter from pmiRGLO and inserting instead minimal promoter sequence. Three variants of mutant plasmids were generated: miPmut1TUT4 (deleted site 1, indicated in new Table S8), miPmut2TUT4 (deleted site 1 and 2, indicated in Table S8) and miPmut3TUT4 (deleted site 1, 2 and 3 indicated in Table S8). For the construction of miPGLO-TUT4, cDNA was prepared from day 5 of HSkM differentiation. PCR was used to amplify the fragments of interest before they were cloned into the pmirGLO Dual-Luciferase miRNA Target Expression Vector (Promega). siRNAs used in this study were synthetized by FutureSynthesis (siMEF2C_1 and siCTRL) or ordered in Dharmacon (SMARTpool siMEF2C_2). siMEF2C_2 SMARTpool consist of 4 siRNAs, 3 of them target mouse Mef2c.The siRNA sequences are listed in Supplementary Table S1.

The fusion index was calculated as the number of nuclei within myotubes expressed as a percentage of the total number of nuclei in the image frame. The images were analyzed using ZEN image analysis module (Zeiss). Calculations were performed for three independent samples from siMEF2C_1 or siMEF2C_2-treated and siCTRL-treated cells.

### RT-PCR and real-time RT-qPCR

Total RNA was extracted with TRIzol reagent (Invitrogen) from cells according to the manufacturer's protocol. Complementary DNA (cDNA) was prepared from 1 μg of total RNA using the GoScript Reverse Transcription System (Promega). cDNA equivalent to 10 ng of the initial RNA input was used as a template for qPCR. For miRNA analysis, cDNA was synthesized in a coupled polyadenylation reverse transcription reaction by using 2 μg of total RNA for 1 h at 37 °C in RT buffer (10 mM Tris–HCl pH 8.0, 75 mM KCl, 10 mM DTT, 70 mM MgCl_2_, 20 U RNasin, 2.5 mM of all four deoxynucleoside triphosphates, 0.5 mM of rATP, and 800 ng of anchored oligo(dT) primer) supplemented with 200 U of Superscript III reverse transcriptase (Invitrogen) and 5 U of *E. coli* poly(A) polymerase (PAP, New England Biolabs). Reactions were heat-inactivated for 10 min at 85 °C. Then, 2 μl of the 9 × diluted cDNA template was used for each qPCR with a reverse primer complementary to the anchored sequence and a probe-specific forward primer (600 nM each)^26^. RT-qPCR was performed using the Power SYBR Green PCR Master Mix (Applied Biosystems, USA), and samples were run in technical triplicates on a 7900HT Fast Real-Time PCR instrument. Ct values were normalized against the internal controls: GAPDH, U6 or miR-16. Fold differences in expression level were calculated according to the 2 − ΔΔCt method. Primers are listed in Supplementary Table S2. Assays to quantify mature miRNAs were performed using a TaqMan MicroRNA Reverse Transcription Kit (Applied Biosystems, Foster City, CA, USA) and an RT-primer containing microRNA-specific stem-loop primers for miR-1, miR-133a, miR-133b, miR-206, miR-16 and U6 (ThermoFisher assay-IDs: 002,222, 002,246, 002,247, 000,510, 000,391 and 001,973).

### Small RNA sequencing and data analysis

Small RNA sequencings were performed two times according to two different protocols. In the first protocol total RNA was purified by 12% PAGE, and a fraction of small RNAs of 10–30 nt in length was excised . Small RNA libraries were generated using a TruSeq Small RNA kit (Illumina) according to the manufacturer’s instructions. Number of biological replicas per group: 3. To equalize the read depth, the libraries were multiplexed with four libraries sequenced per lane of Illumina HiSeq 2000. In the second protocol, 200 ng of total RNA was used to prepare libraries with a NEXTflex Small RNA-Seq Kit (Bioo Scientific). Sequencing parameters: 1 × 50 bp; NovaSeq 6000. In both cases data were processed in the same way. Small RNA-seq data (from Illumina HiSeq 2000 ) were subjected to adapter clipping with an in-house Python script, and read redundancy was removed with a fastx_collapser tool from the FASTX-Toolkit package (http://hannonlab.cshl.edu/fastx_toolkit/). The reads in a FASTA format were then mapped against a set of human pre-miRNA sequences obtained from the miRBase 21 database^42^ using Megablast from the BLAST package, version 2.2.26^43^. It was required that the reads mapped in a sense orientation with no mismatches over the full read length. Then, using an in-house Python script, miRNA expression levels were calculated in the following way: raw counts of all reads mapped to a pre-miRNA sequence in a region occupied by an annotated mature miRNA + / − 2 nt were summed up. The raw expression values served as an input for subsequent differential expression analysis with DESeq2 using default settings and requiring that the adjusted *P* value was below 0.05. To analyze modifications of small RNA fragments, only adapter-containing reads with a length of at least 12 nt were kept. Then, the reads were filtered for quality with a fastq_quality_filter from the FASTX-Toolkit, requiring that a minimum of 95% of nucleotides had a Phred quality score of at least 20. Finally, read redundancy and conversion to the FASTA format were performed with fastx_collapser from the same package. The processed reads were then mapped against human pre-miRNA sequences downloaded from the miRBase 21 database^42^ using Bowtie^44^, allowing for up to 2 mismatches (-v 2 option) and requiring that all found alignments be reported (-a). Using the mapped reads data and known human mature miRNA sequences from miRBase 21, miRNA modifications were identified with miRMOD^45^ using the following settings: (i) the read count threshold was set to 2 from a default of 10,(ii) identification of modifications at both mature miRNA ends was turned on,and (iii) both trimming and nucleotide addition modifications were turned on.

### In vitro transcription and pre-miRNA uridylation assays

DNA templates for pre-let-7a-1, pre-miR-27b and pre-miR-424 were obtained by chemical synthesis and purified by PAGE. Each oligomer contained the T7 RNA polymerase promoter sequence at the 3′-end (Supplementary Table S3). The experimental strategy and protocols for the preparation of DNA templates and in vitro RNA transcripts that are free of detectable 5′-end heterogeneity have been previously described^46^. Pre-miRNAs were incubated in 30 μl reaction mixtures containing 50% (v/v) total HSkM cell extract (approximately 10 μg × μl − 1), 0.5 mM rATP, 20 mM creatine phosphate, and 3.2 mM MgCl_2_. The reactions were supplemented with 0.25 mM rUTP and incubated at 37 °C for 30 min followed by phenol–chloroform extraction, ethanol precipitation, and 8% (w/v) denaturing gel electrophoresis^47^. The bands on the gel were visualized by exposure to a phosphorimaging screen and subsequent scanning on a FLA-5100 scanner (Fujifilm) and were quantified using Multi Gauge software (FujiFilm).

### Western blot analysis

Cell extracts were prepared in 50 mM Tris–HCl pH 7.4, 100 mM NaCl, 1% NP-40, 1 × protease inhibitor (Roche) and 1 mM PMSF. Proteins were separated by Bolt 4–12% Bis–Tris Plus and transferred to a PVDF transfer membrane (0.45 μM, Santa Cruz), and protein detection was carried out with standard western blotting techniques. Rabbit polyclonal anti-TUT4 (18,980–1-AP, Proteintech) was used as the primary antibody, and a secondary, anti-rabbit antibody (A9169; Sigma) was employed (the same anti-TUT4 antibody was used to detect human and mouse protein TUT4). MEF2C primary antibody was purchased from Cell Signaling Technology (D80C1) XP Rabbit mAb #5030. Signals were detected with a Luminata Forte kit (Merck) and quantified with GeneTools from Syngene (https://www.syngene.com/software/genetools-automatic-image-analysis/). To avoid errors related to sample loading, signals for TUT4, MEF2C, and reference protein were obtained from the same separation and the membranes were cut prior to incubation with antibodies.

### Statistical analysis

Statistical significance was determined by Student's *t*-test; ∗ for *P* < 0.05, ∗  ∗ for *P* < 0.01 and ∗  ∗  ∗ for *P* < 0.001. All quantification of the miRNA or gene expression level based on at least three experimental replicas and were performed with GraphPad Prism 8.3.0 (https://www.graphpad.com/). The statistical analysis for linear regression was done by using the GraphPad Prism 8.3.0 tool.

## Supplementary Information

Supplementary Information 1.

Supplementary Information 2.

Supplementary Information 3.

Supplementary Information 4.

## Data Availability

GEO accession numbers: GSE127801, GSE147837 and GSE147838. https://www.ncbi.nlm.nih.gov/geo/query/acc.cgi?acc=GSE127801, https://www.ncbi.nlm.nih.gov/geo/query/acc.cgi?acc=GSE147837, https://www.ncbi.nlm.nih.gov/geo/query/acc.cgi?acc=GSE147838.
