# Supplementary material for: MEF2C shapes the microtranscriptome during differentiation of skeletal muscles
Source: Sci Rep. 2021 Feb 10;11:3476. doi: 10.1038/s41598-021-82706-2 (PMC7875991; doi:10.1038/s41598-021-82706-2)

Supplementary Information
MEF2C shapes the microtranscriptome during differentiation of skeletal muscles

Agnieszka Piasecka[1], Michał Sekrecki[1], Michał Wojciech Szcześniak[2] and Krzysztof Sobczak[1,*]

[1] Laboratory of Gene Therapy, Department of Gene Expression, Institute of Molecular Biology and Biotechnology, Faculty of Biology, Adam Mickiewicz University, Uniwersytetu Poznanskiego 6, 61-614 Poznań, Poland

[2] Institute of Human Biology and Evolution, Faculty of Biology, Adam Mickiewicz University, Uniwersytetu Poznanskiego 6, 61-614 Poznań, Poland

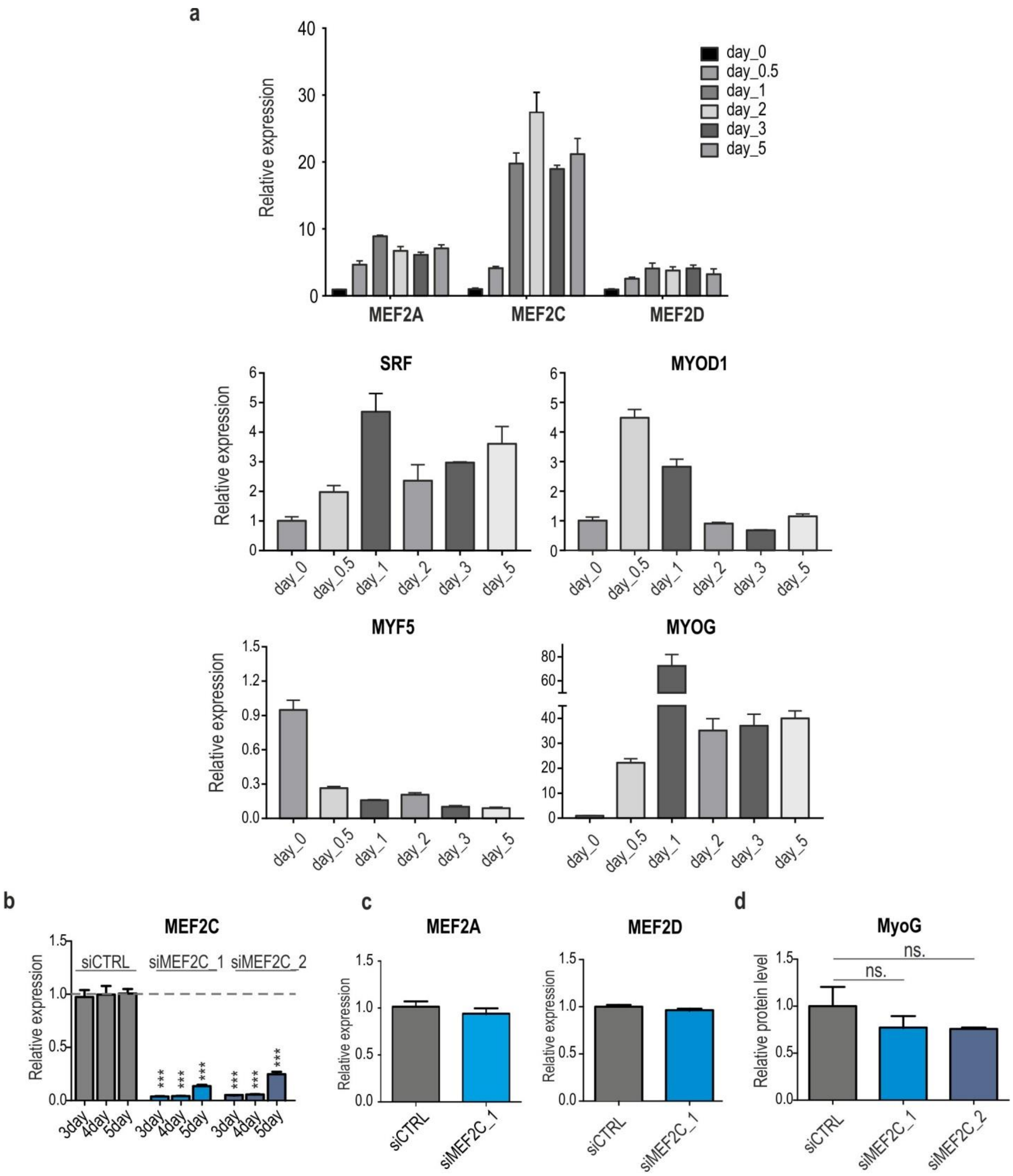

Figure S1. **Expression of muscle-specific genes in the differentiation of HSkMs** (related to **Fig. 1**). (a) Skeletal muscle cells were cultured in growth medium (day 0) as myoblasts and induced to differentiation via changing the medium, and cells were harvested at various time points (12 h, and days 1, 2, 3 and 5) and RNA isolated for the analysis of gene expression. The relative mRNA levels of MEF2s,

SRF, MYOD1, MYF5 and MYOG were calculated based on RT-qPCR assays. Differences in gene expression are shown as fold changes normalized to GAPDH (mean from at least three experiments ± SD). (b) MEF2C knock-down efficiency after 3, 4 and 5 days of differentiation. MEF2C level was determined in cells treated with siCTRL, siMEF2C_1 or siMEF2C. The measurements were normalized to GAPDH (mean from at least three experiments ± SD) (***P < 0.0001). (c) mRNA level of paralogs of MEF2C, namely MEF2A and MEF2D, were not changed upon siRNA mediated MEF2C knock-down. The results were normalized to GAPDH (mean from at least three experiments ± SD). (d) Myogenin protein level upon MEF2C knock-down according to western blot. Signal of MyoG was normalized to Tubulin. (mean from at least three experiments ± SD).

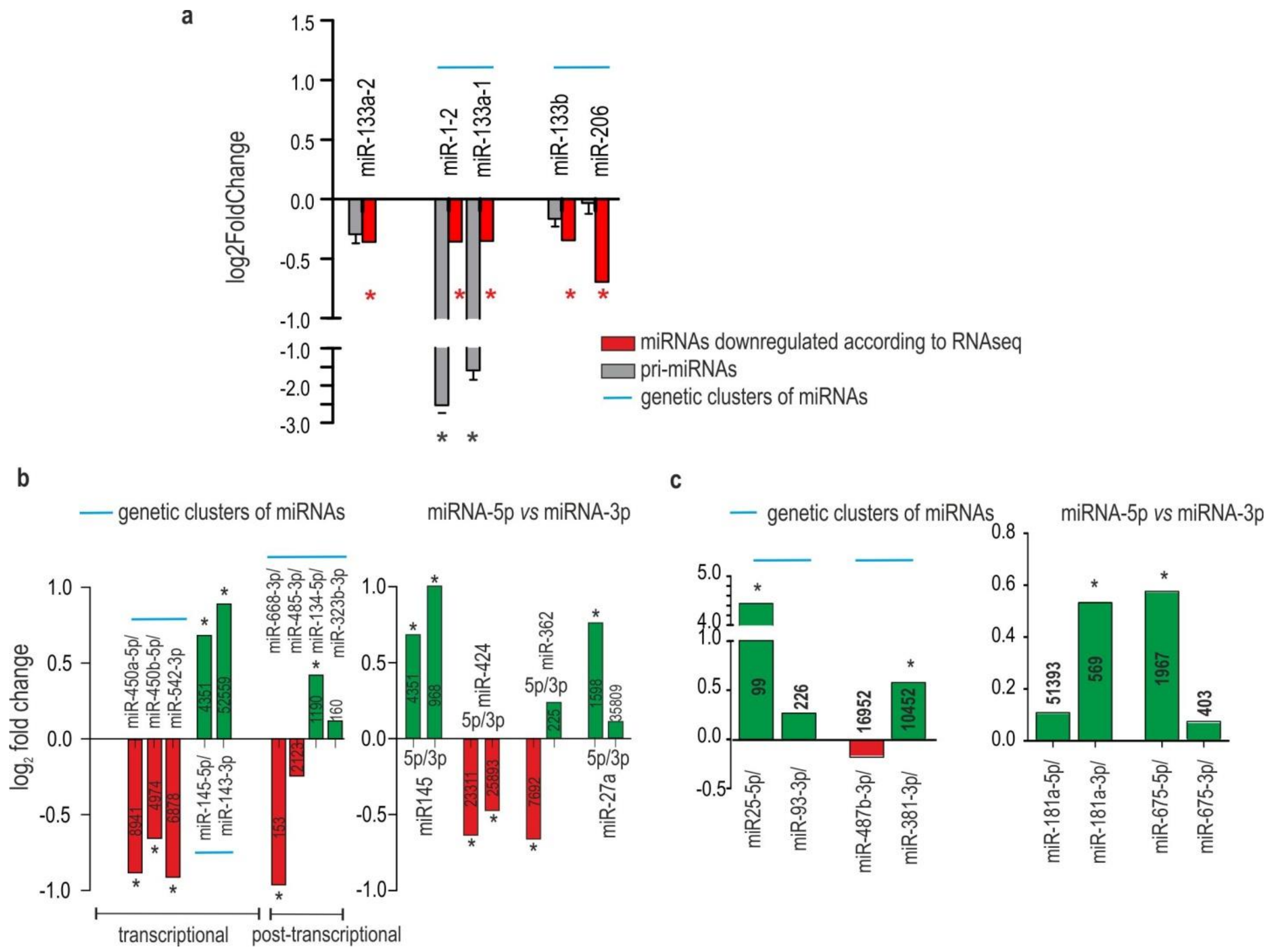

Figure S2. **Changes in the expression level of miRNAs in HSkM cells with MEF2C depletion** (related to **Fig. 2**). (a) Relative expression changes of pri-miRNAs quantified by RT-qPCR assays; all data for pri-miRNA were normalized to GAPDH. Red bars represent miRNAs predicted to be downregulated according to RNA-seq data analysis from siMEF2C_1 treated cells (* Padj < 0.05). (b) Expression changes of miRNAs coming from the same pre-miRNA or from the same genetic clusters. Inside each bar specific value showing miRNAs baseMean count were introduced. (c) Figure 2d with introduced baseMean count value for each miRNA.

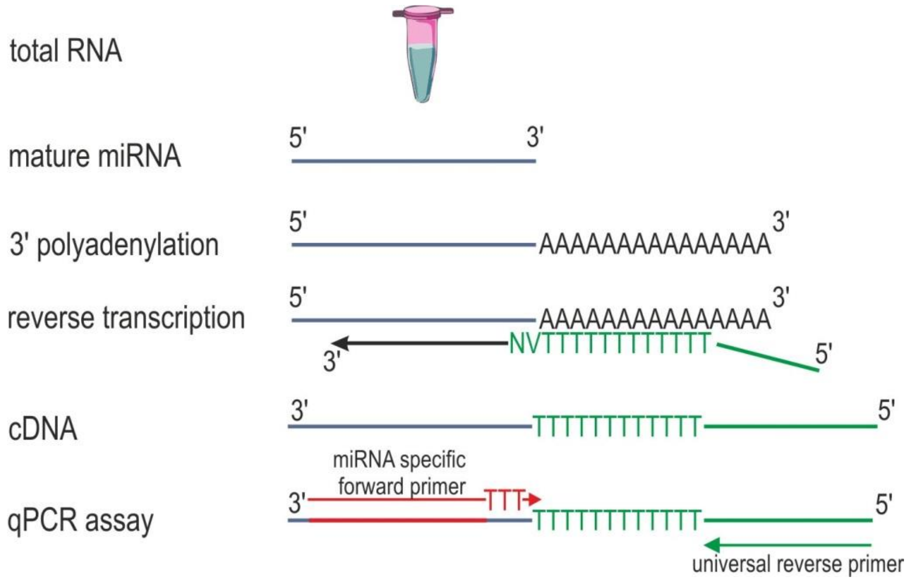

Figure S3. **Scheme of poly(A) tailing-based RT-qPCR modified to distinguish uridylated miRNA isoforms** (related to **Fig. 3**). Total RNA was poly(A) tailed using E. coli poly(A) polymerase. Poly(A)-tailed miRNAs were converted into cDNA in a reverse transcription reaction primed by a standard oligo-dT-anchor adaptor. Then, cDNA was amplified and quantitated by real-time PCR using an miRNA-specific forward primer and a universal reverse primer. The miRNA-specific forward primer is complementary to the 3' end of the miRNA and additionally contains three U residues; therefore, the method allows for preferential amplification of uridylated isoforms of miRNAs.

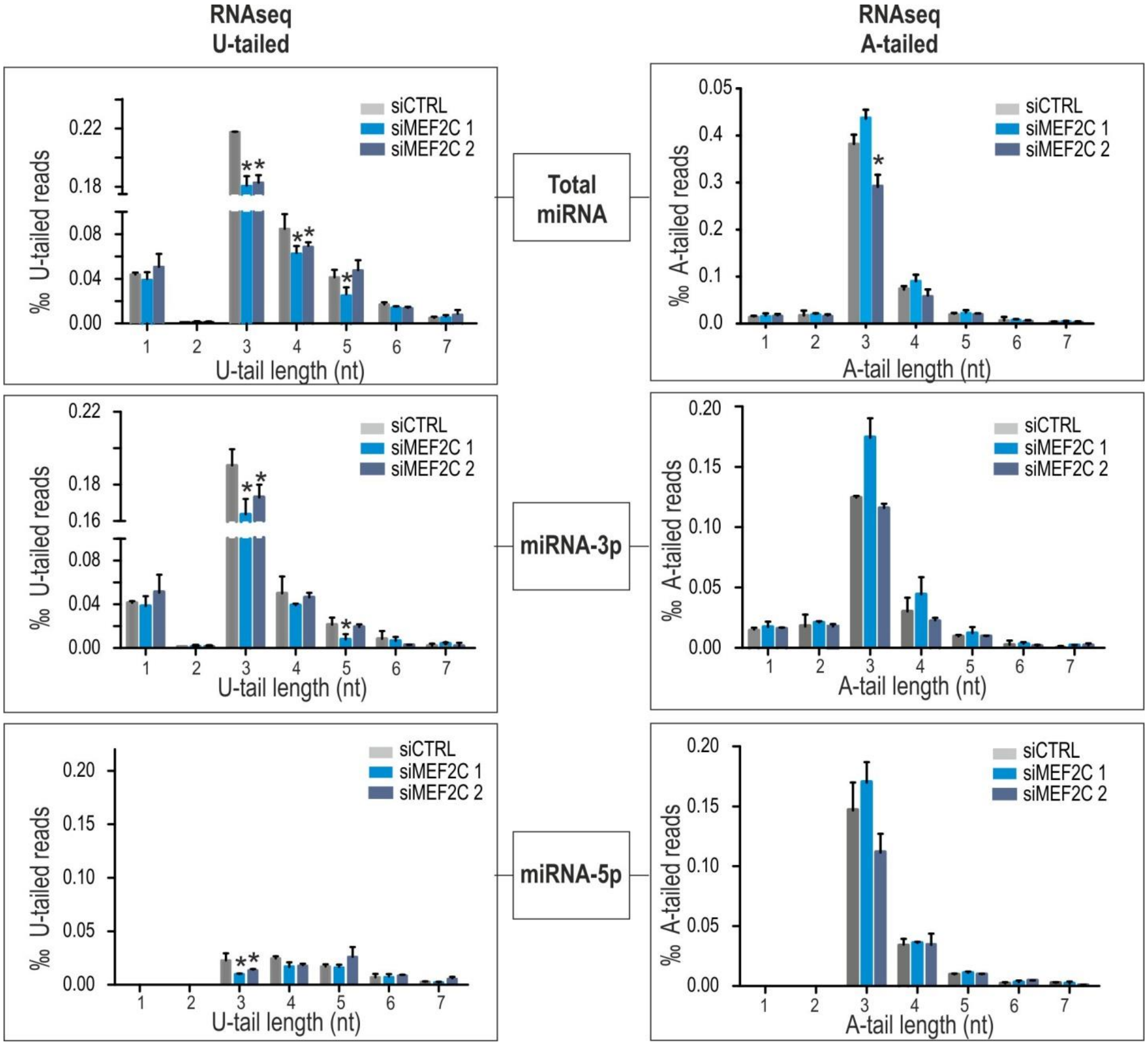

Figure S4. **Comparison of the number and length of U- and A-tailed reads from control and siMEF2C-treated myocytes** (related to **Fig. 3**). Total changes in 3'-nontemplated U- and A-nucleotide additions upon MEF2C depletion are depicted (top graphs). The 3' U- and A-tailed forms were further distinguished by the type of miRNA depends on its location within pre-miRNA (miRNA-3p or miRNA-5p) (*P < 0.05).

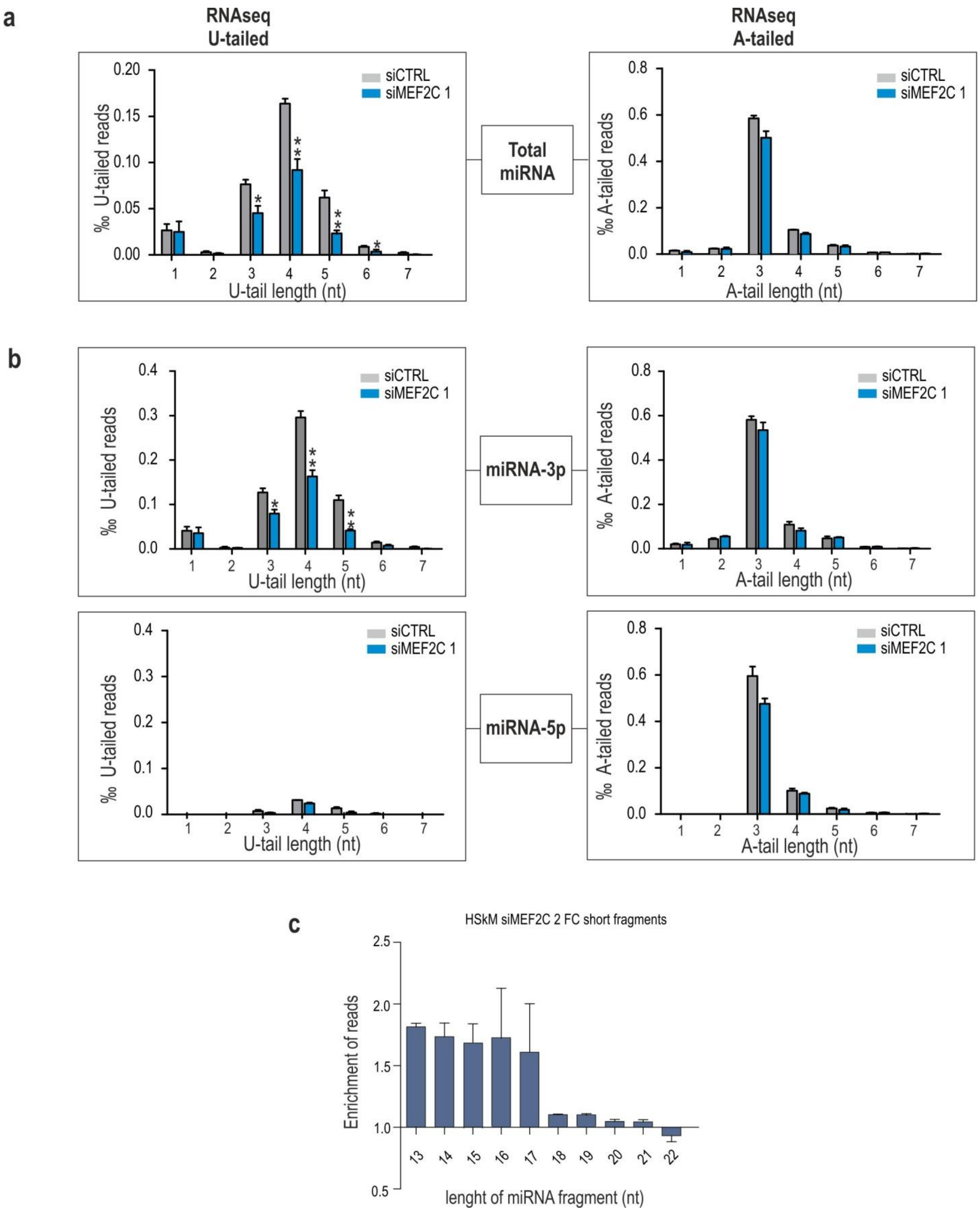

Figure S5. (related to **Fig. 3**)a) **Comparison of the number and length of 3'-nontemplated U- and A-tailed reads of miRNAs in control and siMEF2C_1 treated myocytes**. The X-axis represents the length of U- or A-tails, and the y-axis represents the percentile of modified reads in small RNA-seq. b) The 3' U- and A- forms were further distinguished by the type of miRNA depending on the location of the pre-

miRNA (miRNA-3p or miRNA-5p).The results of TruSeq Small RNA libraries sequencing. c) Accumulation of short fragments of miRNAs in HSkM cells upon siMEF2C_2 depletion in RNA-seq data. Alterations in the level of miRNA fragments are shown as fold changes; fold change is defined as the ratio between the counts of short fragments of miRNA in siMEF2C_2 KD and the counts of short fragments of miRNA in siCTRL cells divided by the counts of short fragments of miRNA in siCTRL cells (no change is equal to1).

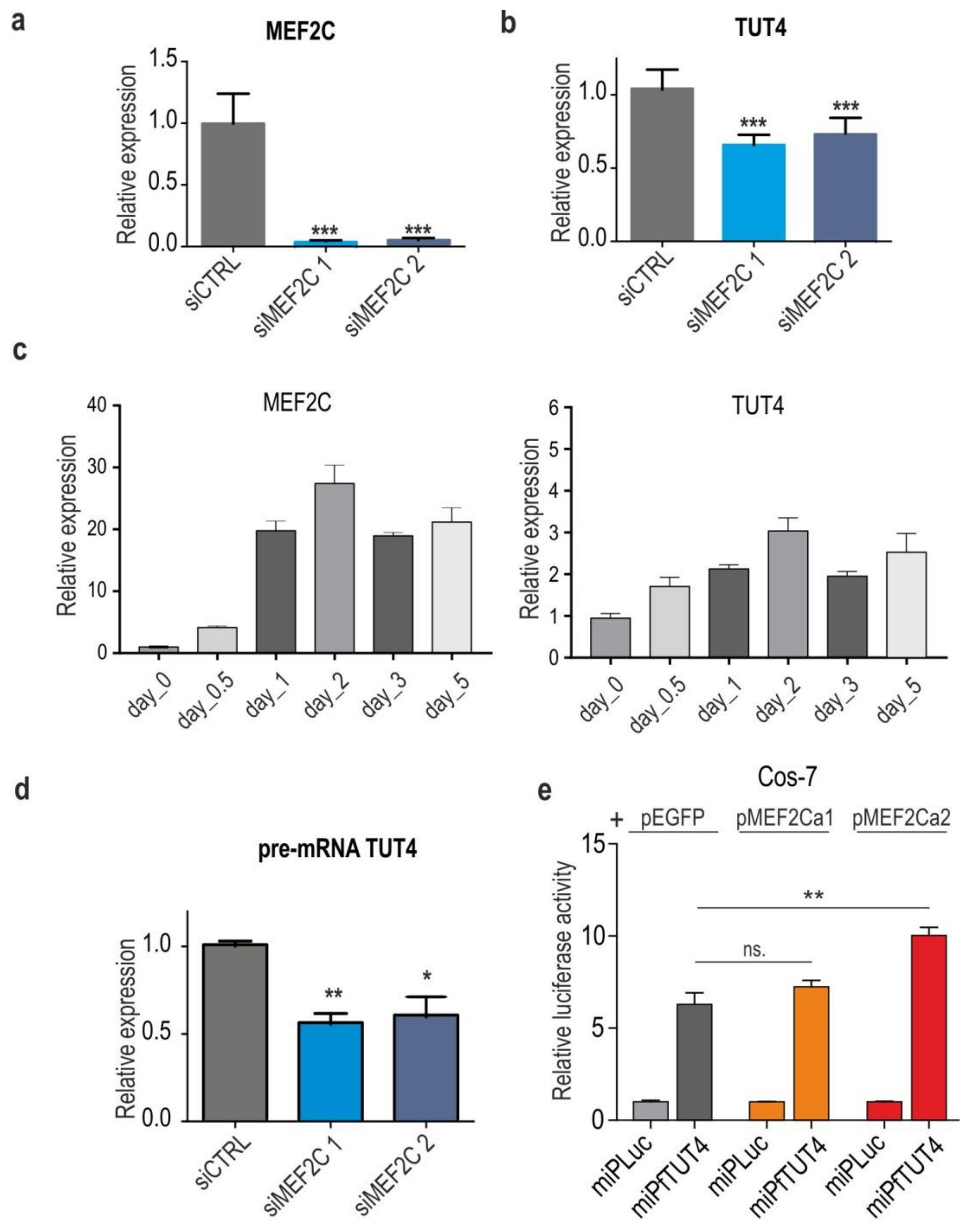

Figure S6. **TUT4 is a target for MEF2C** (related to **Fig. 4**). (a) Quantitative results of RT-qPCR analysis for MEF2C gene expression after treatment with siMEF2C_1 and siMEF2C_2. Results were normalized to GAPDH (***P < 0.0001). (b) Quantitative RT-qPCR analysis of TUT4 expression after treatment with siMEF2C_1 and siMEF2C_2. Significant decrease of steady state level of TUT4 mRNA was observed upon MEF2C knock-down. Results were normalized to GAPDH (***P < 0.0001). (c) TUT4 and MEF2C mRNA expression during human skeletal muscle cell differentiation. The results of RT-qPCR are the averages from three independent experiments normalized to GAPDH. (d) Quantitative RT-qPCR results for pre-mRNA of TUT4 after treatment with siMEF2C_1 and siMEF2C_2. Amplification performed with different pair of primers compare to experiments shown in Fig. 4c (primer Forward located in exon and Reverse in intron of TUT4). Results are averages from 3 experiments (+/- SD) normalized to GAPDH (* P < 0.05, **P < 0.01). (e) Normalized luciferase activity calculated for Cos-7 cells cotransfected with genetic constructs expressing two isoforms of MEF2C (MEF2Ca1 and MEF2Ca2) or GFP and the LUC gene with either a control minimal promotor (miPLuc) or minimal promoter of LUC with upstream fragment of TUT4 gene

(miPfTUT4). Sequence of minimal promotor is depicted in Supplementary Table S4. The experiment was performed in 5 replicates, and the P values were assessed by an unpaired Student's t-test (** $P<0.01$).

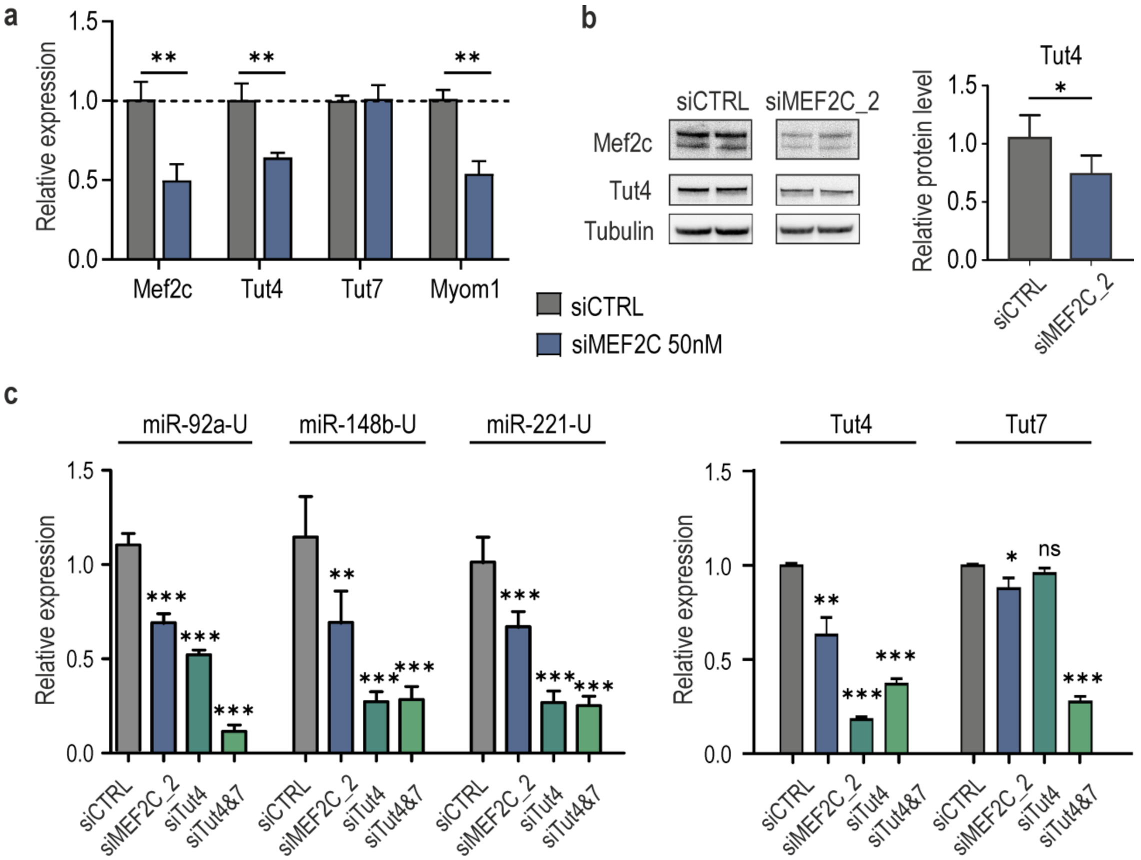

Figure S7. **Mef2c contributes to the 3'-uridylation of miRNAs via TUT4 activity also in mouse myoblasts C2C12** (related to **Fig. 4**). (a) Results of RT-qPCR for mRNAs encoding for two main uridyltransferases (Tut4 and Tut7), Mef2c and its target Myom1; (b) Reduction in the level of TUT4 protein in MEF2C KD conditions confirmed by western blotting. Quantification of TUT4 is depicted in the graph and based on 3 independent experiments normalized to Tubulin (*P<0.05); Signals were quantified with GeneTools from Syngene (https://www.syngene.com/software/genetools-automatic-image-analysis/). Fully uncut gel images are shown in Supplementary information, uncut images section. (c) Modified polyA-RT-qPCR assays were used to quantify uridylated isoforms of miR-221-U, miR-148b-U and miR-92a-U in C2C12 cells with Mef2c deficiency (*P < 0.05, **P < 0.01, ***P < 0.0001). Myoblasts with Tut4 or Tut4 and Tut7 knock-down were used as positive controls.The efficiancy of Tut4 and Tut7 silencing is depicted on the right panel.

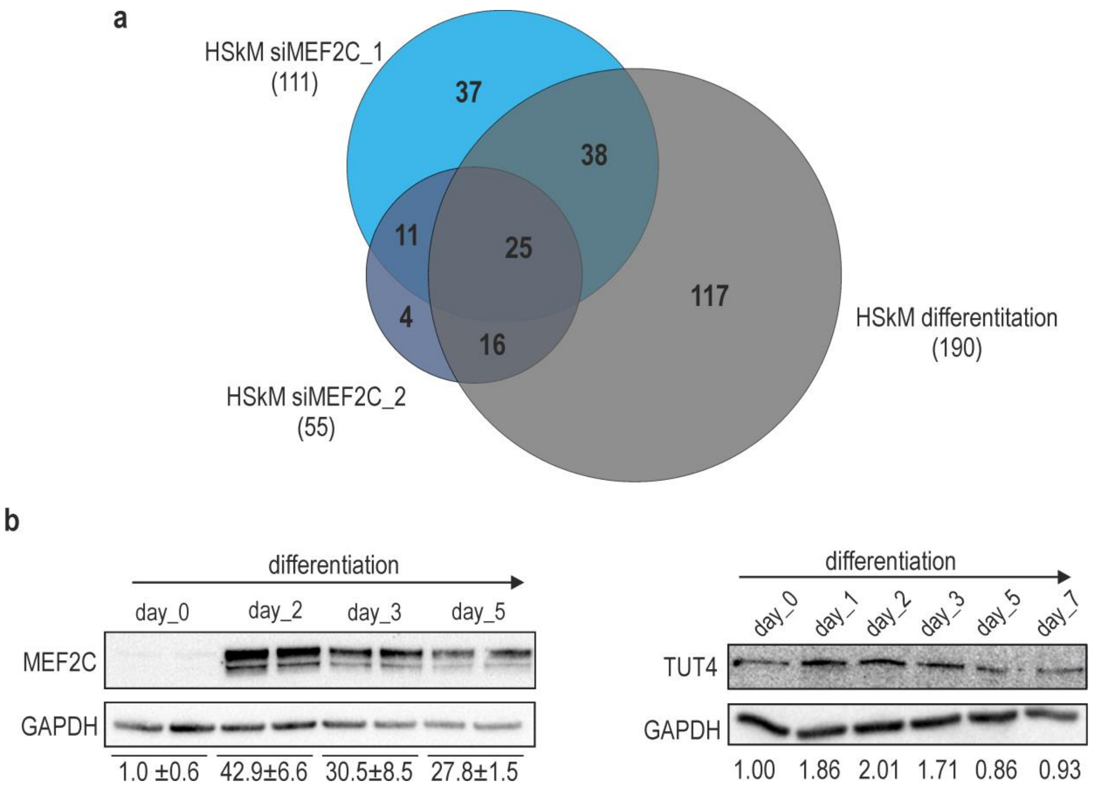

Figure S8. **The overlap of differentially expressed miRNAs in differentiated HSkM cells and in differentiated HSkM cells treated with siMEF2C_1 or siMEF2C_2** (related to **Fig. 5**). (a) Venn diagram illustrates the number of all 190 miRNAs with significantly altered expression in HSkM cells after 4 days of differentiation (HSkM differentiation) and miRNAs significantly changed upon differentiation of MEF2C-deficient HSkMs. Overlapping areas show miRNAs for which expression was significantly altered at two or all data sets (Padj < 0.05) (http://www.biovenn.nl/index.php was used to generate the diagram). (b) MEF2C and TUT4 protein level changes during HSkM differentiation (based on western blot). Quantification of MEF2C and TUT4 is depicted in the panel below western blots. Signals were quantified with GeneTools from Syngene (https://www.syngene.com/software/genetools-automatic-image-analysis/). Fully uncut gel images are shown in Supplementary information, uncut images section.

Supplementary Table S1

siRNAs sequences

siRNA	Sequence (5'>3')
siMEF2C_1_s	P-GACCUGUCAUCUCUGUCUGGGUUUA
siMEF2C_1_as	P-UAAACCCAGACAGAGAUGACAGGUC
siMEF2C_2 SMARTpool	P-GACAAGGAAUGGGAGGAUA<br>P-UAACACAGGUGGUCUGAUG<br>P-GAAUAACCGUAAACCAGAU<br>P-GAUCAGCAGGCAAAGAUUG
siCTRL_s	P-UAAGGCUAUGAAGAGAUACdTdT
siCTRL_as	P-GUAUCUCUUCAUAGCCUUAdTdT

Supplementary Table S2

Name of a primer	Sequence (5’>3’)
MYF5_F	GTGGAGATCCTCAGGAATGC
MYF5_R	AGGTTGCTCTGAGGAGGTGA
MYOD1_F	AGCACTACAGCGGCGACT
MYOD1_R	AGGCAGTCTAGGCTCGACAC
MYOG_F	AAGAGAAGCACCCTGCTCAA
MYOG_R	AGGTTGTGGGCATCTGTAGG
MyHCIIb_F	ATGGCCATGATGACTCACCT
MyHCIIb_R	CTTCCCTGCACCAGATTCTC
SRF_F	GCCACTGGCTTTGAAGAGAC
SRF_R	TCTTCAGCACAGTCCCATTG
MEF2A_F	AGCTCCTCAGAGACCACCAA
MEF2A_R	GGAGGGGGAGACTTTGTAGG
MEF2D_F	GTCATCACTTCCCAGGCAGGAA
MEF2D_R	CGAATGAGTAGACTGGGAGA
MEF2C_F	ATATGCAAGCAAAATCTCCTCC
MEF2C_R	GTTGCTACGGAAAACCACTGGGGTA
mMef2c_F	GTCTGATGGGCGGAGATCTG
mMef2c_R	CTTGCTGCCAGGTGGGATAA
MYOM1_F	TCGTGGTTGAAGAACGAGAA
MYOM1_R	CACCTTTCAGGGACTCCAAG
MYOZ_F	CACAGCTGGTCAGGGATTCT
MYOZ_R	CTGATCCTGTCTCACCAACC
MYOT_F	CAAGAAAGATGCTGGGTGGT
MYOT_R	CGCTGAAATTCTCCTTCTGG
TUT1_F	TCACCCAGAAAGCAGGAGAG
TUT1_R	GGTCAAAAGGGTCCTGGAGA
TUT2_F	CGGAGCAGTGATGGTGATTT
TUT2_R	CAGCACTAACGGACGAACTC
TUT4_F	ACACACTGGATTTTGCTTTGGA
TUT4_R	TTTCCACCCTTCGTCTGAGG
mTut4_F	GGCGTGTCTCACAACTGACT
mTut4_R	GCAAGGAAGAAGAGGGGGTT
mTut7_F	GAGGAGGCACCCAAAGAGAC
mTut7_R	CGATACGCTTTTTGGGCCAG
PARN_F	AAGTGTACCAGGCCATAGAG
PARN_R	CCATTTGTTAATGCAGAGACTG
PNPT1_F	GGATTCAGGGGTTCCAATTT
PNPT1_R	TTTGCCACTGAAGCTTGTTG
Rrp41_F	GGGCCCTAGTGAACTGTCAA
Rrp41_R	CTGCATAGGTCCCACCATCT
DIS3L2_F	GAGCCAGCACAGCAGGTC
DIS3L2_R	CAGGATGGCGCTGTACTTG
TUT4-3’UTR_F	AGATAGCTAGCTCAGCTGGTCTACCGATGC
TUT4-3’UTR_R	AGATATCTAGACAGACAAAATCAACTTTCCATTCA
pre-mRNA TUT4_F	GTTACTCCCCATTCCCCACT

pre-mRNA TUT4_R	GGGAGGGGACATAATCCAGT
pre-mRNA TUT4_F2	GATTCCCGAGATGTTCTTGAC
pre-mRNA TUT4_R2	AGGTTTGCAACAGAGAGGAA
pri-miR-499a_F	GGAGACAGACCCTCCCTCTT
pri-miR-499a_R	GTCTTCACTTCCCTGCCAAA
pri-miR-128_F	CGGTGGAACTCTGACTCCAT
pri-miR-128_R	GTTCGTGCTGCTCTTTGGAT
miR-92a-3p-U	GCACTTGTCCCGGCCTGTTTTT
miR-221-3p-U	CATTGTCTGCTGGGTTTCTTT
miR-362-5p	AATCCTTGGAACCTAGGTGT
miR-499a-5p	TTAAGACTTGCAGTGATGTT
miR-16	AGCAGCACGTAAATATTGGC
U6	GGATGACACGCAAATTCGTG
miR-22-3p	AAGCTGCCAGTTGAAGAACT
miR-1	TGGAATGTAAAGAAGTATGT
miR-21	AGCUUAUCAGACUGAUGUUG
miR-206	TGGAATGTAAGGAAGTGTGT
miR-133a	TGGTCCCCTTCAACCAGCTG
miR-133b	TGGTCCCCTTCAACCAGCTA
miR-92a	TATTGCACTTGTCCCGGCCT
miR-221-3p	CTACATTGTCTGCTGGGTTTC
miR-500a	ATGCACCTGGGCAAGGATTC
miR-376a-3p	ATCATAGAGGAAAATCCACG
miR-376c-3p	AACATAGAGGAAATTCCACG
miR-483-3p	TCACTCCTCTCCTCCCGTCT
miR-7-5p	GGAAGACTAGTGATTTTGTT
miR-378a	ACTGGACTTGGAGTCAGAAG
miR-148a	TCAGTGCACTACAGAACTTT
miR-532	CATGCCTTGAGTGTAGGACC
miR-361	TTATCAGAATCTCCAGGGGT
miR-27a-5p	AGGGCTTAGCTGCTTGTGAGC
TUT7_F	GGAAGCCACGGAAGACTAGA
TUT7_R	CTGCAGGTGTACTTTGCTGT
pri-miR-22_F	GCAGAGGGCAACAGTTCTTC
pri-miR-22_R	CAGCGAGGTTAACAGCTTCC
pri-miR-483_F	GGAACCACTCCCTTCTTTCC
pri-miR-483_R	GTGAAATGGGCTCACAGGAT
pri-miR-378a_F	TGTTGAAGATTGAACCGAGATGT
pri-miR-378a_R	GTTCCCAGACCATGCCAGT
pri-miR-128_F	ATCCTGATGGCCCTTAGTCA
pri-miR-128_R	TGCCAAGCTGTTACAAACCA
pri-miR-500a_F	TTGGGTGATGAGACTTGCAC
pri-miR-500a_R	GATGTATTTGGGGCCTAGCA
pri-miR-130a_F	AGGGTTCTGTAGTCTTGGGC
pri-miR-130a_R	AGGACAGGACCCACTAAAGC
pri-miR-1-1_F	GGCAGTAGACTCCAGGGAAG
pri-miR-1-1_R	GACACAGGCAAAGTGACAGAAC
pri-miR-1-1_F2	AGAGCTTGAGGGAAACTCCAC
pri-miR-1-1_R2	ATCATCAGCAACGCTGACTC
pri-miR-133a-2_F	AGAGCTTGAGGGAAACTCCAC

pri-miR-133a-2_R	GACAGTCGATTTGGTTCCATTT
pri-miR-1-2_F	GCAAAAAGAATCAAACCAGGAC
pri-miR-1-2_R	CATTCCATAGCATTGTATGTTCA
pri-miR-1-2_F2	CTCATCCTGGTTTTTCTCCTTG
pri-miR-1-2_R2	AAGCATGCAGAAAGTCATAAGC
pri-miR-133a-1_F	GCAGGAAAACAGTAGGAAAGTG
pri-miR-133a-1_R	ACAAATGAAAACGTTGGTTGTC
pri-miR-206_F	TCCCAGTGATCTTCTCGCTAAG
pri-miR-206_R	GGAGATAGGGGTGTTCAGGAAG
pri-miR-133b_F	ACACACCAAGATACCTGCACAC
pri-miR-133b_R	TGACTCCAGGACTCCTCTTCTC
pri-miR-23a_F	ATGGGATTTGCTTCCTGTCA
pri-miR-23a_R	ACTTGGTGTGGACCCTGCT
pri-miR-27a_F	AGGGCTTAGCTGCTTGTGAG
pri-miR-27a_R	CAAACCAACTGTGTTTCAGCTC
pri-miR-7-2_F	TCCAGGGAATTCTGAGGTGA
pri-miR-7-2_R	GGCTGGCACCATTAGGTAGA
pri-miR-7-3_F	CAGGTGAGAAGGAGGAGCTG
pri-miR-7-3_R	TTGTTTGCTTCCCTCCTTTG
pri-miR-222_F	TGCCCAATAATCTCTCTCAGG
pri-miR-222_R	ACCCTCAATGGCTCAGTAGC
pri-miR-7-1_F	GCTGCATTTTACAGCACCAA
pri-miR-7-1_R	GCCATGGTGTCTCAACCTTT
pri-miR-362_F	ACACAAAAAGGGCAGGTGTC
pri-miR-362_R	GTCCAGGGCAATGATGAAGT
pri-miR-376c_F	AAAGTCGGTGGACCTCAGAA
pri-miR-376c_R	AGAAGGTTTCATCCCGGAAA
minimal promotor	AGAGGGTATATAATGGAAGCTCGACTTCCAG

Supplementary Table S3

DNA templates for *in vitro* transcription

**microRNA precursor**	**Sequence of a microRNA precursor (5’>3’)**
pre-let7aT7	GAAAGACAGTAGATTGTATAGTTATCTCCCAGTGGTGGGTGTGACCCTAAAACTATACAACCTACTACCTCCTATAGTGAGTCGTATTA
pre-miR-27bT7	GCAGAACTTAGCCACTGTGAACAAAGCGGAAACCAATCACTGTTCACCAATCAGCTAAGCTCCTATAGTGAGTCGTATTA
pre-miR-424T7	ATAGCAGCGCCTCACGTTTTGAACCATTTAGAACACTTCAAAACATGAATTGCTGCCTATAGTGAGTCGTATTA

Supplementary Table S7

The putative binding sites for MEF2C localized within 1 kb genomic regions spanning each of the top 16 differentially expressed miRNAs in MEF2C deficient myocytes. The table gather the list of best occurrences in each input promoter, with gene name, oligo score (from 0 to 1), position with respect to the transcription start site of the gene ("Position" column), putative binding sequence and strand.

MIR NAME	Gene Name	Score	Position	Sequence	Strand
miR-378a	hg38_refGene_NR_029870	0.920157	28	TACCTAGAAATAGCA	+
miR-376c	hg38_refGene_NR_029861	0.900028	27	TAACCATAAATAACA	-
miR-30d	hg38_refGene_NR_029599	0.897296	-334	AGTTTCAAAATAGTA	-
miR-500a	hg38_refGene_NR_030224	0.894999	-706	TACATAAAAATAGAG	+
miR-486-2	hg38_refGene_NR_106984	0.88298	-258	AGGATAAAATTAGAC	-
miR-532	hg38_refGene_NR_030241	0.881182	-188	GGAATAAAAATAAAG	+
miR-7-2	hg38_refGene_NR_029606	0.878932	-396	TTACTAAAAAAAAAA	+
miR-30a	hg38_refGene_NR_029504	0.872389	-892	CATCTAAAAACAAAT	+
miR-128-2	hg38_refGene_NR_029824	0.842474	-281	GATCAAAAAATAATT	-
miR-486-1	hg38_refGene_NR_030161	0.826622	-534	AAGCCAGAAAGAAAA	+
miR-483	hg38_refGene_NR_030158	0.825365	-721	TTTATATAATTAAAA	+
miR-376a-1	hg38_refGene_NR_029868	0.810732	-775	AACACAAAAGTAACA	-
miR-499a	hg38_refGene_NR_030223	0.801897	-890	CTTCTAACAATGGCA	-
miR-22	hg38_refGene_NR_029494	0.799839	-215	TGGCTGTAAACAGAC	-
miR-139	hg38_refGene_NR_029603	0.78962	-640	AGGACAAGGATAGCT	+
miR-133a-2	hg38_refGene_NR_029676	0.766324	-674	TACCCAGAGATACCA	+

Supplementary Table S8

Variants of mutated miPTUT4 plasmids, which have deleted putative MEF2C binding sites. Three distinct putative binding sites, within upstream region of TUT4, are marked with colors. Mutant miPmut1TUT4 has deleted site 1, marked in the first row of table with red; miPmut2TUT4 mutant has deleted site 1 and 2, marked in the first row of table with red and blue; miPmut3TUT4 mutant has deleted site 1, 2 and 3 marked in the first row of table with red, blue and green, respectively.

Construct name	**TUT4 upstream sequence introduced into the plasmids**
miPTUT4	CAGCCAGATTGGGGTAAAATGCACTAACTCTGAACCCTAGTTTTTTCATCCGTAAATGAGAGCAACAGTAC<br>GTAACTCACAGGGCTATCGTGAAGTGGAGCGTCCCTCTGTGACCTCTAACCAGTCCCAGCCTGAGTCACAT<br>TATAAGAAAA**TTTATAAGGCACTATCTTGTAAATA**CTTAAGATTTAACATCTTCCCTTCACCTCTACGTCC<br>ACTCTTTTCATACCTTTTTCCTGCCTAGCATGTGTCAAACTCAAAGGACTGGGCACAGAGCACAGAGGTTG<br>TGTGCTTTGTCCAGCTTTGAGGTCAGAGATTGG**CTAAATAAT**CTCTGAGGCTGGG**GTCTTAAAAAAAAACA**<br>**ACAAAAAAACAAAACTAACA**AAACCTTAAGTCCGTTCCGCCCGTGACTTTAAATAATTATGTAGAGGAATGT<br>AATACGTTAAAAGCAAGGGGATGAGGGGGCCCATTGATGGTAGATGGTTTTATGCCCGCCTGCAGCTAAAG<br>AAATGATTTCTAAAACCTCTTTTGGGGGTGGAGATGGAGTGCTCCAGAGATTTCTCTTCGAGCTCCTTCA<br>TTCCTGAACTGCCTGCCTTTTCAAAGGAAAAAAGGTCCAGCTGCGGTGGGCTGGAGGCCGTGGTGAGTGGG<br>TGGGGGTCGACCCTGGCGGCTTCCTCTGCCCCGCTCCCTACCGTTGCTCCGAAGCCCGCTTGGGCTTCCGA<br>GGGTCCGCCGAGGCTCGGGCCACGCCCAGTCCGAGTGCCAGGCCAGAGGGTACCCATTCCAGGCCGGCCTT<br>CCTCCCCAGGGGGAGCAGCCTTGGGAGCGAGGCGCCGAGGCGGCCCTCGGGCTCGGCTTCCAGCTCGGGGG<br>CCTCGGCAGAGACTAGGAACCAGACTGGGGCAGCACTGGGGCGCCCGCCACCCCCACCCACTCCGGCAAGC<br>GGGCGAATCAGCGCGGCTAACCCGCGGCGCCCAGGCGGGCTGCGGGACCTCGCGACCTCCGCTCGGCGGCC<br>CCGCCCCTGCCCGCGAGCCCCGGGTGGCTCCCTCCTCCCCTCTGCCAGCGGCCCGCCCCC
miPmut1TUT4	CAGCCAGATTGGGGTAAAATGCACTAACTCTGAACCCTAGTTTTTTCATCCGTAAATGAGAGCAACAGTAC<br>GTAACTCACAGGGCTATCGTGAAGTGGAGCGTCCCTCTGTGACCTCTAACCAGTCCCAGCCTGAGTCACAT<br>TATAAGAAAA**TTTATAAGGCACTATCTTGTAAATA**CTCAAGATTTAACATCTTCCCTTCACCTCTACGTCC<br>ACTCTTTTCATACCTTTTTCCTGCCTAGCATGTGTCAAACTCAAAGGACTGGGCACAGAGCACAGAGGTTG<br>TGTGCTTTGTCCAGCTTTGAGGTCAGAGATTGG**CTAAATAAT**CTCTGAGGCTGGAAACCTTAAGTCCGTTC<br>CGCCCGAAACCTTAAGTCCGTTCCGCCCGGTAGAGGAATGTAATACGTTAAAAGCAAGGGGATGAGGGGGC<br>CCATTGATGGTAGATGGTTTTATGCCCGCCTGCAGCTAAAGAAATGATCTCTAAAACCTCTTTTGGGGGTG<br>GGAGATGGAGTGCTCCAGAGATTTCTCTTCGAGCTCCTTCATTCCTGAACTGCCTGCCTTTTCAAAGGAAA<br>AAAGGTCCAGCTGCGGTGGGCTGGAGGCCGTGGTGAGTGGGTGGGGGTCGACCCTGGCGGCTTCCTCTGCC<br>CCGCTCCCTACCGTTGCTCCGAAGCCCGCTTGGGCTTCCGAGGGTCCGCCGAGGCTCGGGCCACGCCCAGT<br>CCGAGTGCCAGGCCAGAGGGTACCCATTCCAGGCCGGCCTTCCTCCCCAGGGGGAGCAGCCTTGGGAGCGA<br>GGCGCCGAGGCGGCCCTCGGGCTCGGCTTCCAGCTCGGGGGCCTCGGCAGAGACTAGGAACCAGACTGGGG<br>CAGCACTGGGGCGCCCGCCACCCCCACCCACTCCGGCAAGCGGGCGAATCAGCGCGGCTAACCCGCGGCGC<br>CCAGGCGGGCTGCGGGACCTCGCGACCTCCGCTCGGCGGCCCCGCCCCTGCCCGCGAGCCCCGGGTGGCTC<br>CCTCCTCCCCTCTGCCAGCGGCCCGCCCCC
miPmut2TUT4	CAGCCAGATTGGGGTAAAATGCACTAACTCTGAACCCTAGTTTTTTCATCCGTAAATGAGAGCAACAGTAC<br>GTAACTCACAGGGCTATCGTGAAGTGGAGCGTCCCTCTGTGACCTCTAACCAGTCCCAGCCTGAGTCACTC<br>GGGCGGAACGGACCTCAAGATTTAACATCTTCCCTTCACCTCTACGTCCACTCTTTTCATACCTTTTTCCT<br>GCCTAGCATGTGTCAAACTCAAAGGACTGGGCACAGAGCACAGAGGKTGKGTGCTTTGTCCAGCTTTGAGG<br>TCAGAGATTGG**CTAAATAAT**CTCTGAGGCTGGAAACCTTAAGTCCGTTCCGCCCGAAACCTTAAGTCCGTT<br>CCGCCCGGTAGAGGAATGTAATACGTTAAAAGCAAGGGGATGAGGGGGCCCATTGATGGTAGATGGTTTTA<br>TGCCCGCCTGCAGCTAAAGAAATGATCTCTAAAACCTCTTTTGGGGGTGGAGATGGAGTGCTCCAGAGAT<br>TTCTCTTCGAGCTCCTTCATTCCTGAACTGCCTGCCTTTTCAAAGGAAAAAAGGTCCAGCTGCGGTGGGCT<br>GGAGGCCGTGGTGAGTGGGTGGGGGTCGACCCTGGCGGCTTCCTCTGCCCCGCTCCCTACCGTTGCTCCGA<br>AGCCCGCTTGGGCTTCCGAGGGTCCGCCGAGGCTCGGGCCACGCCCAGTCCGAGTGCCAGGCCAGAGGGTA<br>CCCATTCCAGGCCGGCCTTCCTCCCCAGGGGGAGCAGCCTTGGGAGCGAGGCGCCGAGGCGGCCCTCGGGC<br>TCGGCTTCCAGCTCGGGGGCCTCGGCAGAGACTAGGAACCAGACTGGGGCAGCACTGGGGCGCCCGCCACC<br>CCCACCCACTCCGGCAAGCGGGCGAATCAGCGCGGCTAACCCGCGGCGCCCAGGCGGGCTGCGGGACCTCG<br>CGACCTCCGCTCGGCGGCCCCGCCCCTGCCCGCGAGCCCCGGGTGGCTCCCTCCTCCCCTCTGCCAGCGGC<br>CCGCCCCC
miPmut3TUT4	CAGCCAGATTGGGGTAAAATGCACTAACTCTGAACCCTAGTTTTTTCATCCGTAAATGAGAGCAACAGTAC<br>GTAACTCACAGGGCTATCGTGAAGTGGAGCGTCCCTCTGTGACCTCTAACCAGTCCCAGCCTGAGTCACTC

	GGGCGGAACGGACCTCAAGATTTAACATCTTCCCTTCACCTCTACGTCCACTCTTTTCATACCTTTTTCCT<br>GCCTAGCATGTGTCAAACTCAAAGGACTGGGCACAGAGCACAGAGGKTGKGTGCTTTGTCCAGCTTTGAGG<br>TCAGAGATTGGTCTGAGGCTGGAAACCTTAAGTCCGTTCCGCCCGAAACCTTAAGTCCGTTCCGCCCGGTA<br>GAGGAATGTAATACGTTAAAAGCAAGGGGATGAGGGGGCCCATTGATGGTAGATGGTTTTATGCCCGCCTG<br>CAGCTAAAGAAATGATCTCTAAAACCTCTTTTGGGGGTGGGAGATGGAGTGCTCCAGAGATTTCTCTTCGA<br>GCTCCTTCATTCCTGAACTGCCTGCCTTTTCAAAGGAAAAAAGGTCCAGCTGCGGTGGGCTGGAGGCCGTG<br>GTGAGTGGGTGGGGGTCGACCCTGGCGGCTTCCTCTGCCCCGCTCCCTACCGTTGCTCCGAAGCCCGCTTG<br>GGCTTCCGAGGGTCCGCCGAGGCTCGGGCCACGCCCAGTCCGAGTGCCAGGCCAGAGGGTACCCATTCCAG<br>GCCGGCCTTCCTCCCCAGGGGGAGCAGCCTTGGGAGCGAGGCGCCGAGGCGGCCCTCGGGCTCGGCTTCCA<br>GCTCGGGGGCCTCGGCAGAGACTAGGAACCAGACTGGGGCAGCACTGGGGCGCCCGCCACCCCCACCCACT<br>CCGGCAAGCGGGCGAATCAGCGCGGCTAACCCGCGGCGCCCAGGCGGGCTGCGGGACCTCGCGACCTCCGC<br>TCGGCGGCCCCGCCCCTGCCCGCGAGCCCCGGGTGGCTCCCTCCTCCCCTCTGCCAGCGGCCCGCCCCC

Full length un-edited gel image of figures: 1a, 1d, 3e, 4b, S7b, S8b

Full gel images for PCR results show in Figure **1a**. Fragments presented in Fig. **1a** are indicated by red rectangles.

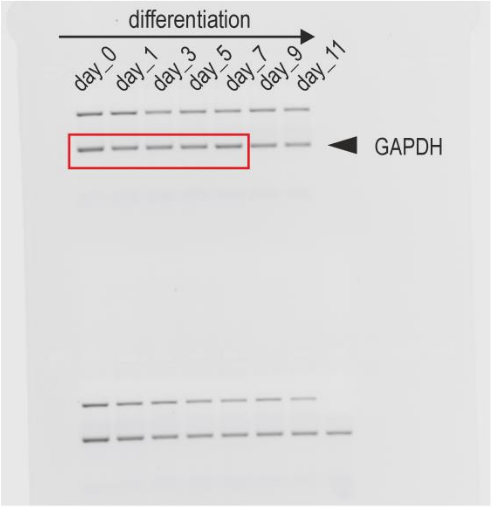

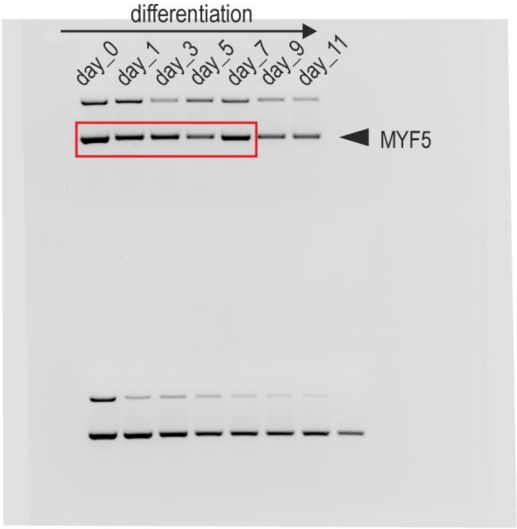

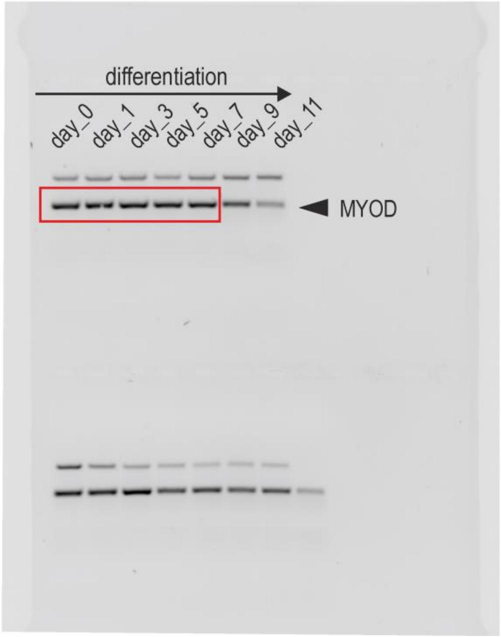

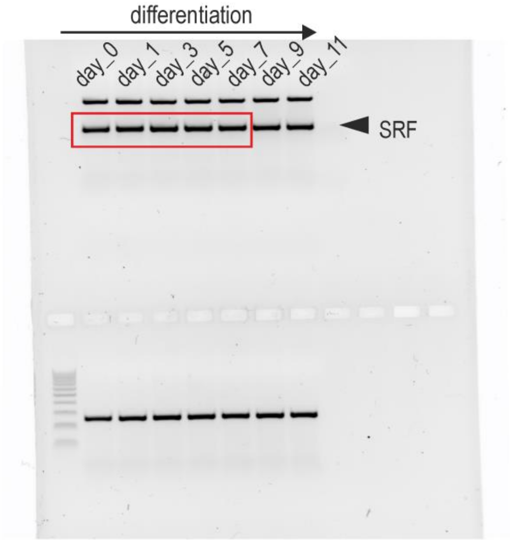

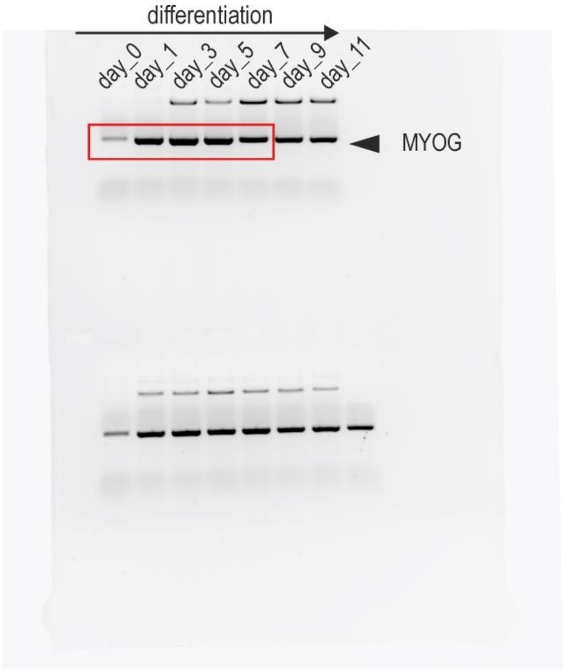
differentiation
day_0
day_1
day_3
day_5
day_7
day_9
day_11
MYOG

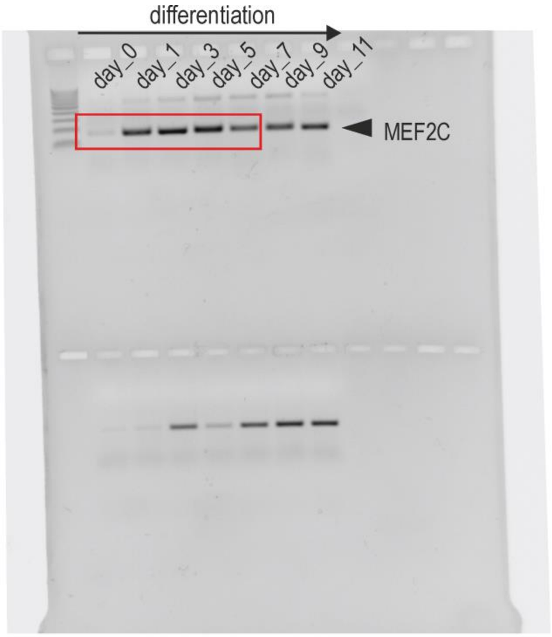
differentiation
day_0
day_1
day_3
day_5
day_7
day_9
day_11
MEF2C

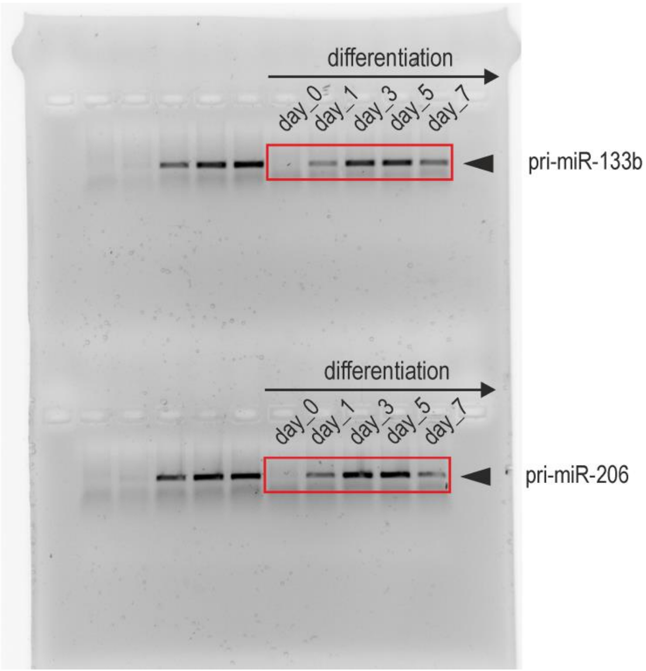
differentiation
day_0
day_1
day_3
day_5
day_7
pri-miR-133b
differentiation
day_0
day_1
day_3
day_5
day_7
pri-miR-206

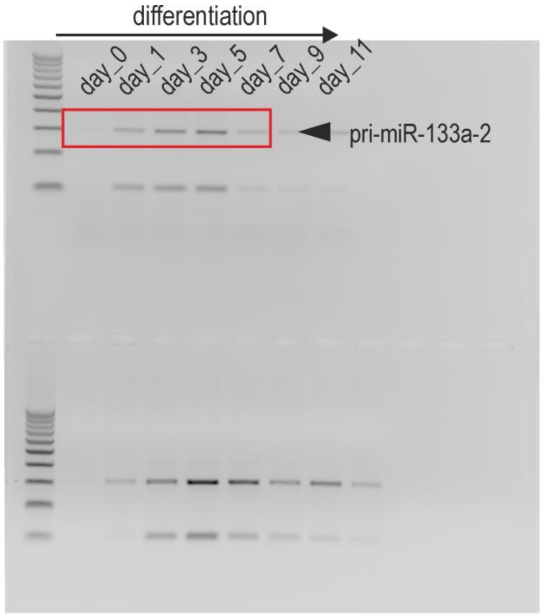
differentiation
day_0
day_1
day_3
day_5
day_7
day_9
day_11
pri-miR-133a-2

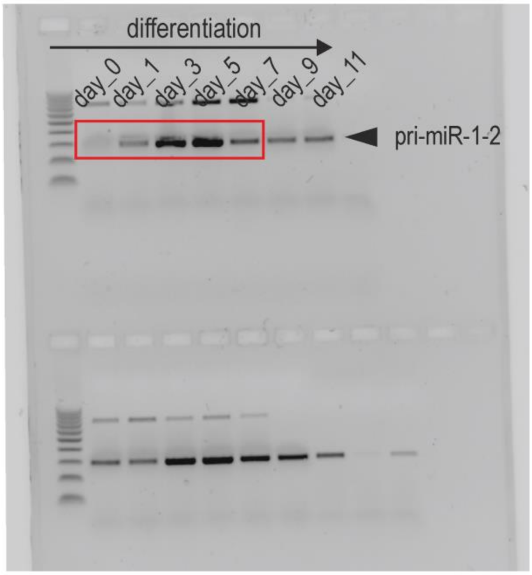
differentiation
day_0
day_1
day_3
day_5
day_7
day_9
day_11
pri-miR-1-2

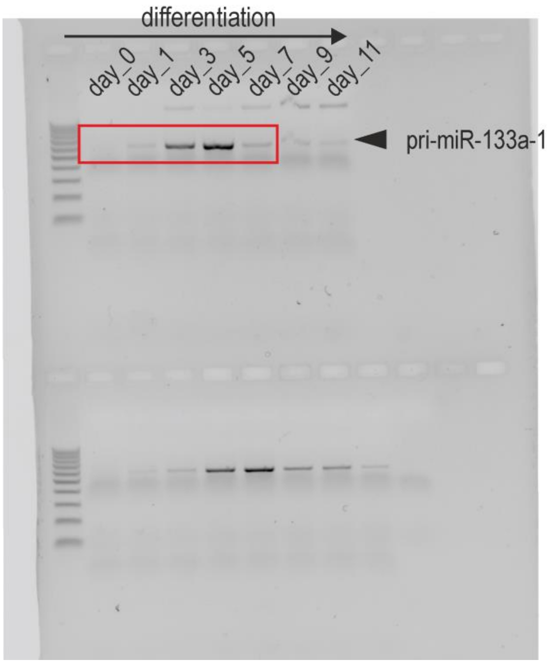
differentiation
day_0
day_1
day_3
day_5
day_7
day_9
day_11
pri-miR-133a-1

Full gel images for western blot results show in Figure **1d**. Fragments presented in Fig. **1d** are indicated by red rectangles.

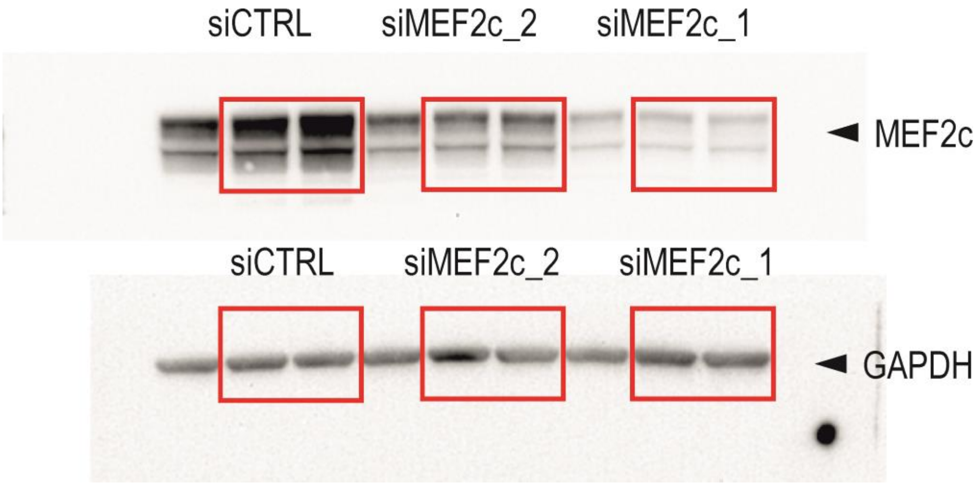

Full gel image for uridylation assay results show in Figure **3e**. Fragment presented in Fig. **3e** is indicated by red rectangle.

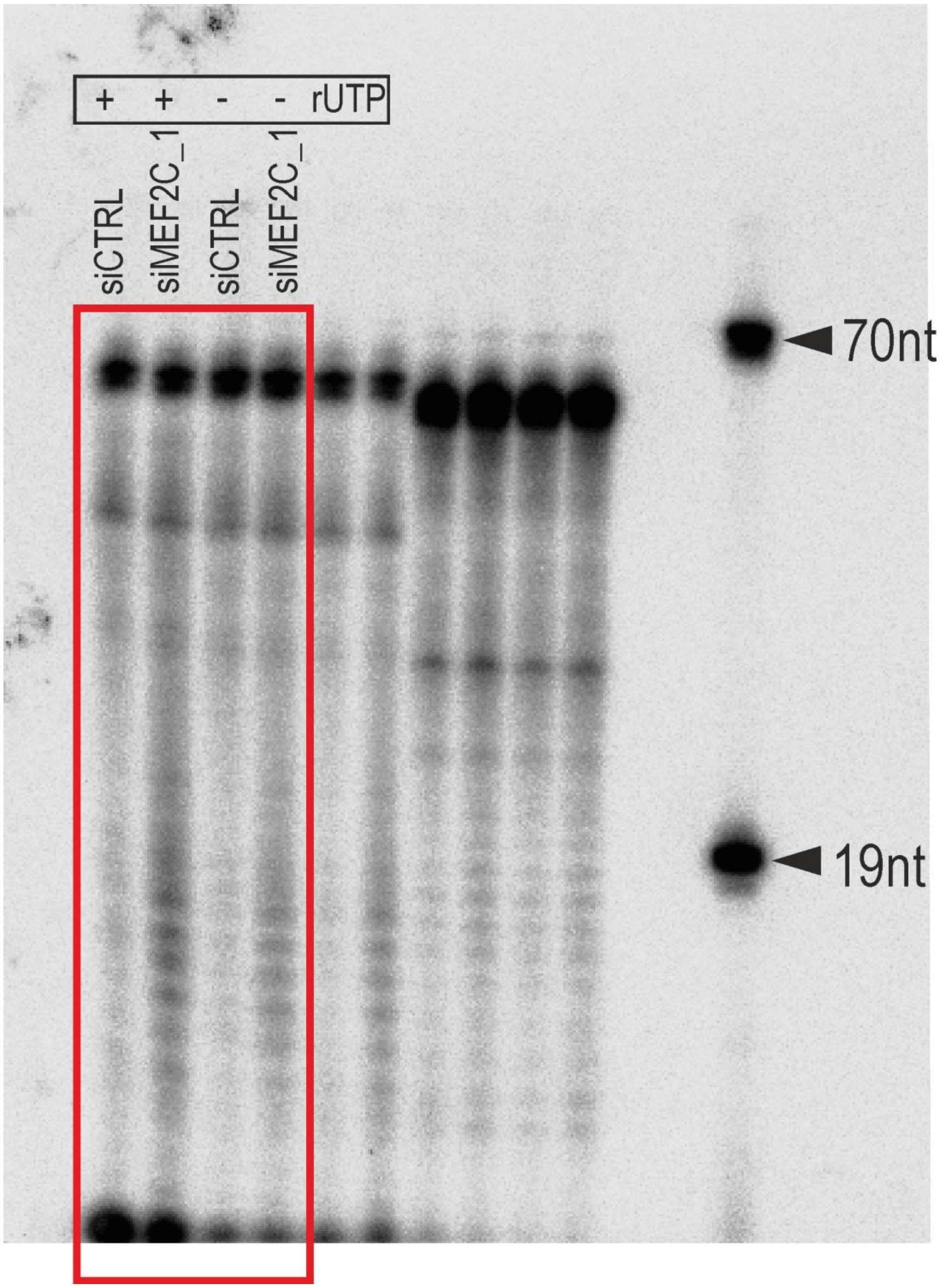

Full gel images for western blot results show in Figure **4b**. Fragments presented in Fig. **4b** are indicated by red rectangles.

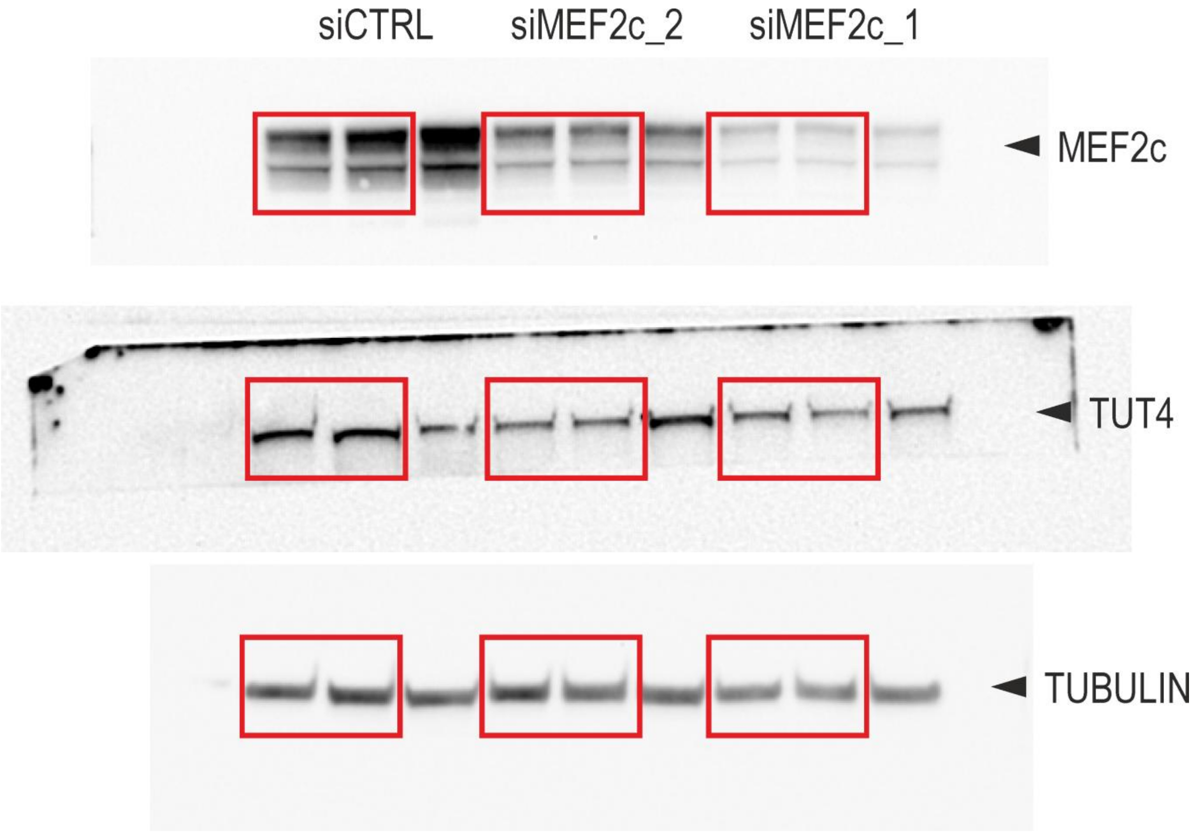

Full gel images for western blot results show in Figure **S7b**. Fragments presented in Fig. **S7b** are indicated by red rectangles. Additional full gel images were taken with visible molecular weight size marker (M).

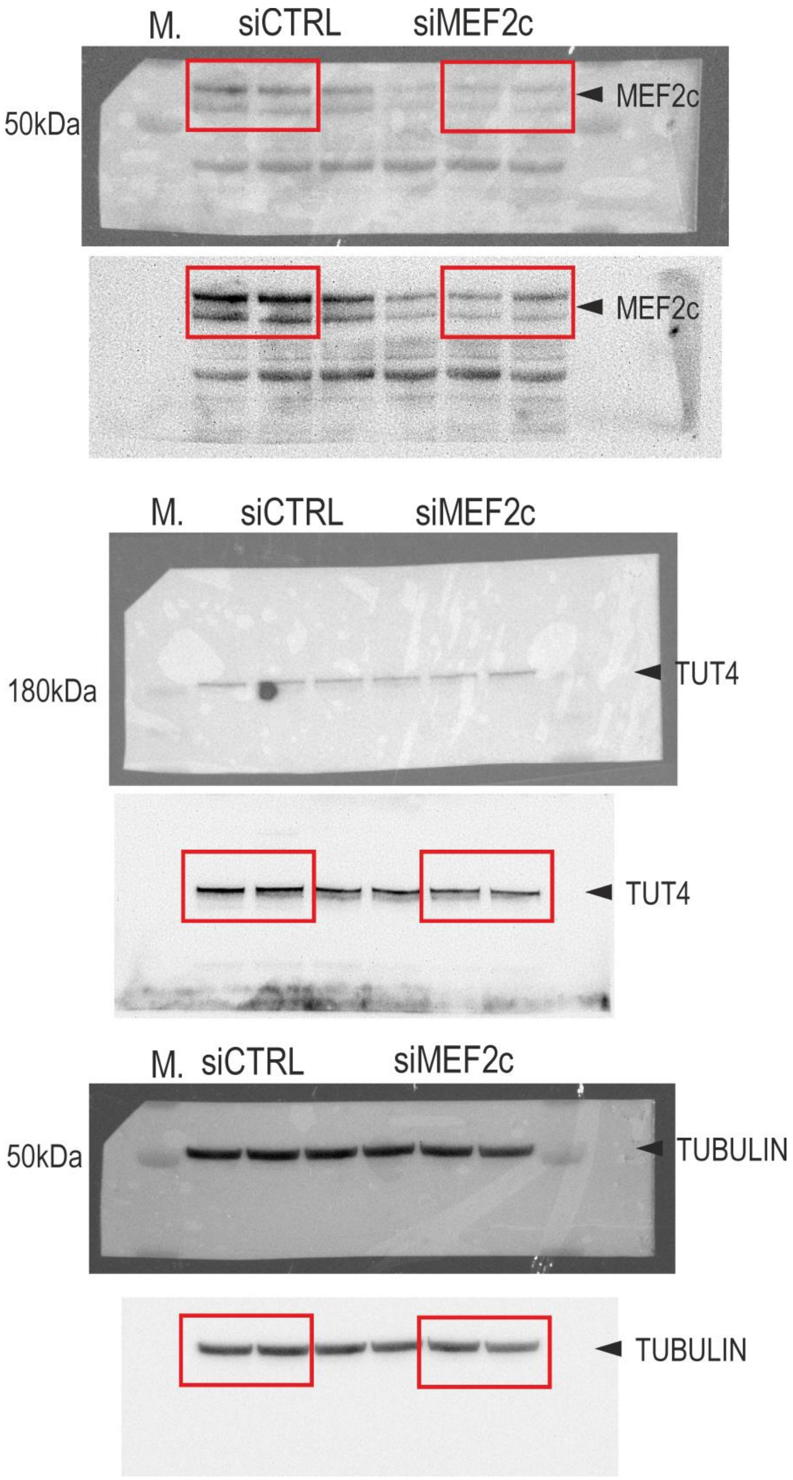

Full gel images for western blot results show in Figure **S8b**. Fragments presented in Fig. **S8b** are indicated by red rectangles. Additional full gel images were taken with visible molecular weight size marker (M).

Fig.S8b, right panel

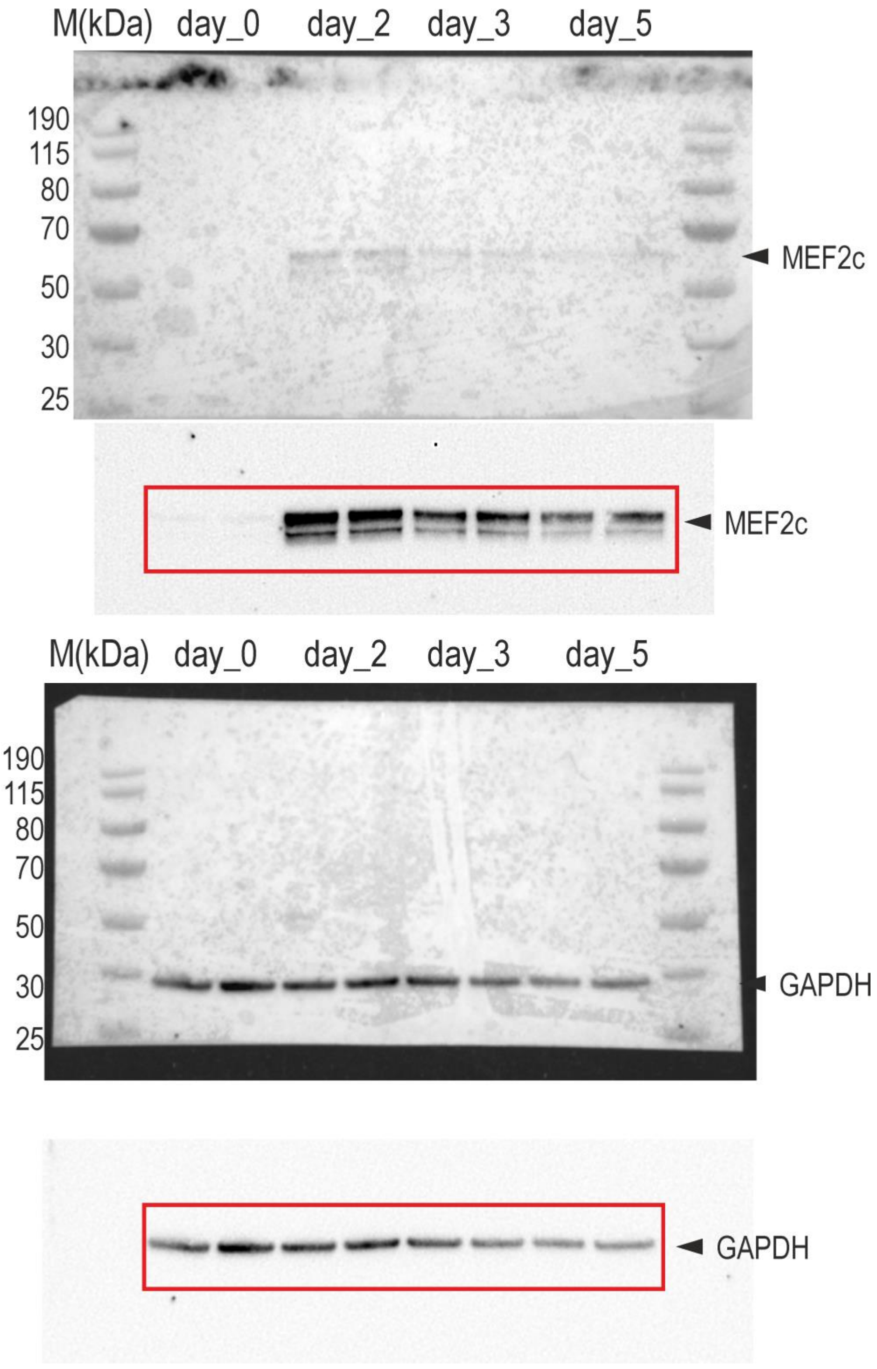

Fig.S8b, left panel

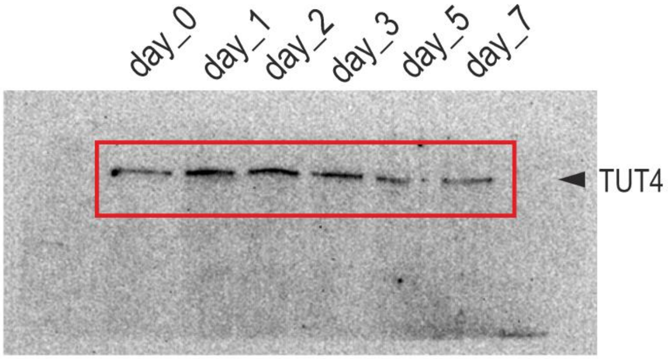

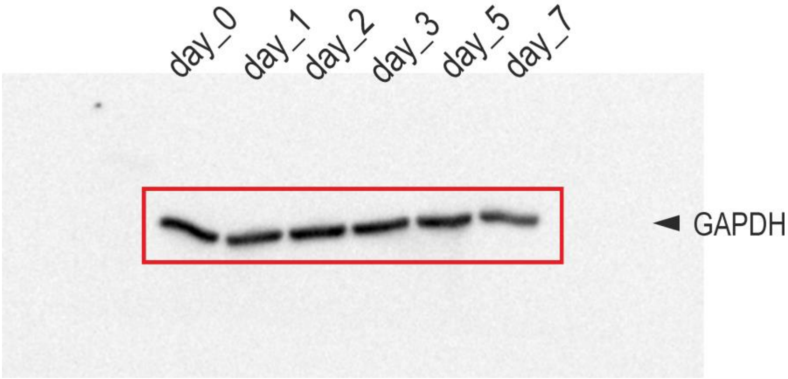

Supplement: Supplementary file 4 — Supplementary Information 4. [file 41598_2021_82706_MOESM4_ESM.pdf]
